# Time-scale dynamics of proteome and transcriptome of the white-rot fungus *Phlebia radiata*: growth on spruce wood and decay effect on lignocellulose

**DOI:** 10.1186/s13068-016-0608-9

**Published:** 2016-09-05

**Authors:** Jaana Kuuskeri, Mari Häkkinen, Pia Laine, Olli-Pekka Smolander, Fitsum Tamene, Sini Miettinen, Paula Nousiainen, Marianna Kemell, Petri Auvinen, Taina Lundell

**Affiliations:** 1 Microbiology and Biotechnology, Department of Food and Environmental Sciences, University of Helsinki, P.O.Box 56, Viikki Biocenter 1, 00014 Helsinki, Finland; 2 DNA Sequencing and Genomics Laboratory, Institute of Biotechnology, University of Helsinki, Helsinki, Finland; 3 Proteomics Unit, Institute of Biotechnology, University of Helsinki, Helsinki, Finland; 4 Laboratory of Organic Chemistry, Department of Chemistry, University of Helsinki, Helsinki, Finland; 5 Laboratory of Inorganic Chemistry, Department of Chemistry, University of Helsinki, Helsinki, Finland

**Keywords:** Wood decay, White-rot, Proteomics, Transcriptomics, *Phlebia radiata*, Phlebioid, Lignin biodegradation, Lignin-modifying enzymes, Carbohydrate-active enzymes, Peroxidases

## Abstract

**Background:**

The white-rot Agaricomycetes species *Phlebia radiata* is an efficient wood-decaying fungus degrading all wood components, including cellulose, hemicellulose, and lignin. We cultivated *P. radiata* in solid state cultures on spruce wood, and extended the experiment to 6 weeks to gain more knowledge on the time-scale dynamics of protein expression upon growth and wood decay. Total proteome and transcriptome of *P. radiata* were analyzed by peptide LC–MS/MS and RNA sequencing at specific time points to study the enzymatic machinery on the fungus’ natural growth substrate.

**Results:**

According to proteomics analyses, several CAZy oxidoreductase class-II peroxidases with glyoxal and alcohol oxidases were the most abundant proteins produced on wood together with enzymes important for cellulose utilization, such as GH7 and GH6 cellobiohydrolases. Transcriptome additionally displayed expression of multiple AA9 lytic polysaccharide monooxygenases indicative of oxidative cleavage of wood carbohydrate polymers. Large differences were observed for individual protein quantities at specific time points, with a tendency of enhanced production of specific peroxidases on the first 2 weeks of growth on wood. Among the 10 class-II peroxidases, new MnP1-long, characterized MnP2-long and LiP3 were produced in high protein abundances, while LiP2 and LiP1 were upregulated at highest level as transcripts on wood together with the oxidases and one acetyl xylan esterase, implying their necessity as primary enzymes to function against coniferous wood lignin to gain carbohydrate accessibility and fungal growth. Majority of the CAZy encoding transcripts upregulated on spruce wood represented activities against plant cell wall and were identified in the proteome, comprising main activities of white-rot decay.

**Conclusions:**

Our data indicate significant changes in carbohydrate-active enzyme expression during the six-week surveillance of *P. radiata* growing on wood. Response to wood substrate is seen already during the first weeks. The immediate oxidative enzyme action on lignin and wood cell walls is supported by detected lignin substructure sidechain cleavages, release of phenolic units, and visual changes in xylem cell wall ultrastructure. This study contributes to increasing knowledge on fungal genetics and lignocellulose bioconversion pathways, allowing us to head for systems biology, development of biofuel production, and industrial applications on plant biomass utilizing wood-decay fungi.

**Electronic supplementary material:**

The online version of this article (doi:10.1186/s13068-016-0608-9) contains supplementary material, which is available to authorized users.

## Background

Lignocellulosic biomass is a large renewable resource of carbon that can be used as a substrate in the production of biofuels and biochemicals contrary to the polluting and diminishing fossil hydrocarbon sources. In nature, the carbon from lignocellulosic substrates including wood is utilized and recycled by fungi, most belonging to wood-colonizing and litter-decomposing Basidiomycota of the class Agaricomycetes [1–3]. The wood-decaying Polyporales species have been divided into white-rot and brown-rot fungi based on their visually observable decay types and differences in carbohydrate-active enzyme (CAZyme) encoding gene repertoires [1, 4]. Especially, the white-rot fungi are interesting due to their ability to degrade all components of wood, including the recalcitrant, aromatic, and heterogeneous lignin polymers [2, 5].

Lignin degradation is an important step prior to industrial use of plant biomass and lignocellulose raw materials [6]. In the white-rot fungal lifestyle—before gaining access to the carbohydrate storages of cellulose and hemicelluloses—lignin barrier is attacked to facilitate utilization of these carbon and energy sources [2, 7]. Lignin modification is possible because of a wide array of extracellular oxidoreductases produced by white-rot fungi. These oxidoreductases are enzymes of CAZy auxiliary activity family 2 (AA2) [8] fungal class-II lignin-modifying peroxidases including lignin peroxidases (LiPs), manganese peroxidases (MnPs), and versatile peroxidases (VPs) that are important in lignin modification [2, 9, 10]. Class-II peroxidases require hydrogen peroxide which may be generated by other CAZy auxiliary activity enzymes belonging to copper radical oxidases (CROs, AA5) and glucose–methanol–choline superfamily (GMCs, AA3) oxidoreductases [11, 12]. Lignin-converting enzyme set also includes the dye-decolorizing peroxidases (DyPs) [10, 13] and laccases. Laccases are phenol-oxidizing multicopper oxidases (MCOs, CAZy class AA1) which may thereby potentially act on lignin substructures by the aid of aromatic mediator compounds [2, 5].

Crystalline cellulose is utilized by the white-rot fungi with the help of cellobiohydrolases belonging to CAZy glycoside hydrolase (GH) families GH6 and GH7 [1, 3]. For complete enzymatic degradation of cellulose chains, endoglucanases of several GH families (GH5, GH9, GH12, GH44, and GH45), family AA9 (GH61) lytic polysaccharide monooxygenases (LPMO), and β-glucosidases of families GH1 and GH3 are needed [2, 14]. In addition, Basidiomycota genomes encode a wide array of other carbohydrate-active enzymes such as carbohydrate esterases (CEs) and polysaccharide lyases (PLs) for degradation of wood components including hemicelluloses and pectins [14]. The genes expressed and proteins produced during growth on plant biomass material reflect specific lifestyle of each fungal species and its strategy utilized for lignocellulose conversion [2, 15–17].

*Phlebia radiata* is a saprobic, wood-colonizing white-rot species of Agaricomycetes order Polyporales and phlebioid clade, and it is the taxonomic type species of the genus *Phlebia* [18, 19]. In nature, *Phlebia* species are mainly found colonizing deciduous wood and to some extent, also on coniferous wood [20, 21]. *P. radiata* and other *Phlebia* species are able to grow on Norway spruce (*Picea abies*) wood, producing wood-decaying enzymes [18, 22]. Spruce and coniferous wood from northern temperate and boreal forests are significant renewable feedstocks for forest-based industry [23]. To investigate the applicability of *P. radiata* isolate 79 for wood pre-treatment and lignocellulose bioconversions, we selected Norway spruce as its growth substrate for the proteomic and transcriptomic analyses.

Several lignin-modifying enzymes of *P. radiata* 79 were previously cloned and characterized, including three LiPs [24], two divergent MnPs [25], and two laccases [26, 27]. Especially, the lignin-modifying peroxidases (LiPs and MnPs) of *P. radiata* and near-related *Phlebia* isolates have demonstrated high activity and efficiency in oxidoreductive reactions, conversion and degradation of lignin-like molecules, and potential in biotechnological applications [28–31]. However, no complete proteomic or transcriptomic study of the fungus on its natural lignocellulose wood substrate has been conducted before.

Our aim was to analyze the time-dependent changes in protein and enzyme expression of *P. radiata* during 6 weeks of growth on wood under conditions mimicking the natural fungal habitat. Transcriptome analysis from two cultivation time points served as a support for the proteomics study and also provided additional information on gene expression during growth on wood. The genome assembly of *P. radiata* (to be discussed elsewhere) was functionally annotated and searched for CAZyme encoding genes which were upregulated and produced as proteins on spruce wood.

## Results

Genome sequencing of *P. radiata* wild-type dikaryon isolate 79 resulted with 40.92-Mb haploid size genome assembly including 14,113 predicted gene models (to be discussed elsewhere). To study the proteome of *P. radiata*, to recognize as many proteins as possible, and to identify time-dependent expression of lignocellulose degrading CAZymes on coniferous wood, total proteins from six time points (0, 7, 14, 21, 28, 42 days of growth) were extracted from solid-state spruce wood cultivations. In addition to proteomics, transcriptome on wood at growth time points of 14 and 28 days was compiled by RNA sequencing to facilitate analysis of differential gene expression by using the 14-day malt extract medium grown mycelia as reference.

### Characteristics of *P. radiata* proteome and transcriptome on wood

In total, 1356 proteins were identified by peptide LC–MS/MS proteomics and mapping the peptide sequences against translated coding sequences of the gene models of *P. radiata* genome assembly (with at least two unique peptides mapping per protein, Additional file 1: Table S1). For each protein at each time point, the mean abundance value with standard deviation was calculated from the three biological replicate culture values (Additional file 1: Table S1). The biological replicate protein abundances had high coherence according to principal component analysis (Additional file 2: Figure S1a). The number of identified proteins increased up to 28 days then slightly decreased on day 42 (Table 1). This was in accordance with total protein concentrations measured from protein extracts of each time point (Additional file 3: Figure S2).

**Table 1:** Number of identified proteins in the proteome, and proteins with N-terminal secretion signal sequence at the six time points of *P. radiata* cultivation on spruce wood

Time (d)	Number of identified proteins^a^	Number of secreted proteins^b^ (percentage of identified proteins)
0	89	31 (35 %)
7	1009	150 (15 %)
14	1077	163 (15 %)
21	1149	170 (15 %)
28	1151	170 (15 %)
42	1035	157 (15 %)

The proteins were analyzed by peptide LC–MS/MS and identified by searching against translated gene models of *P. radiata* genome. The secreted proteins were recognized with Phobius prediction analysis [32]

^a^Proteins were identified from peptide LC–MS/MS analysis

^b^Secreted proteins were identified with Phobius prediction

**Fig. 1:**
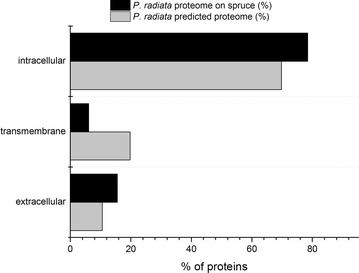
Cellular localization of the proteins of *P. radiata* identified in the proteome on spruce wood and predicted from the translated protein models annotated on the genome assembly. Analysis was performed computationally

To estimate the number of secreted proteins in the total proteome, Phobius analysis [32] was performed. N-terminal signal peptide was predicted for 15 % (210) of the proteins (Fig. 1). This percentage was higher than the number of secreted proteins according to the in silico analysis of *P. radiata* gene models (10 %, Fig. 1). The number of secreted proteins may be an underestimation due to difficulties in recognition of the true 5’ initiation site (start Met codon) of gene model ORFs by computational methods. However, in silico analysis gave a rough estimation of the ratio of the number of theoretically predicted versus proteomics-obtained number of secreted proteins.

**Fig. 2:**
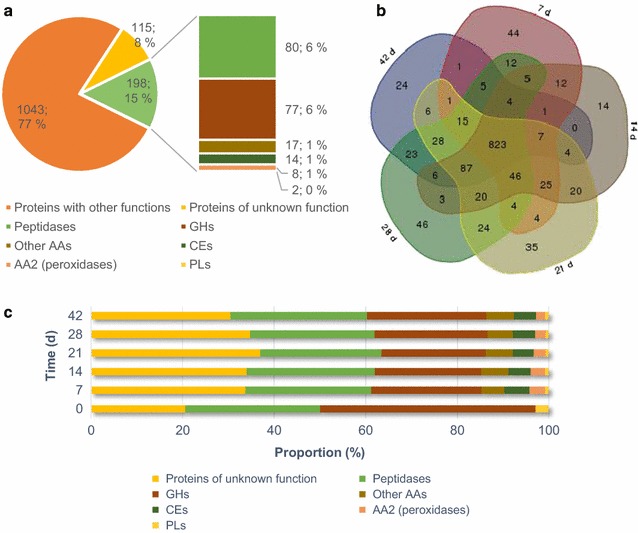
Functional distribution of proteins identified in *P. radiata* proteome on spruce wood by LC–MS/MS peptide analysis. **a** Distribution of the total identified proteins (1356) into functional classes. **b** Venn diagram of distribution of identified proteins (1349) in the fungal proteomes on wood extracted from five weekly time points. **c** Distribution in percentage of wood-decay CAZy and other proteins at the six time points. Proteins with equal or more than two unique peptides were included in the analyses

Overall, composition of *P. radiata* proteome was relatively constant in the course of spruce wood solid-state cultivation (Fig. 2b, c). In total, 823 (61 %) proteins were shared at each time point from day 7 to 42. As expected, we identified some proteins (89) at time point zero representing those introduced to the solid wood substrate within the fungal inoculum (cultivated on malt extract liquid medium for 14 days). Blast2GO searches showed that the identified proteins were divided into various functional categories (Fig. 2a). Based on the preliminary annotation of the gene models, proteins were divided into eight different categories: AA2 (class-II peroxidases), other AAs, CEs, GHs, peptidases, PLs, proteins with other functions, and proteins of unknown function. The majority of identified proteins (77 %) were classified as proteins with other functions that include an array of intracellular proteins involved in translation and in metabolic processes.

The transcriptomes of two biological replicate cultures on spruce wood at two time points (14 and 28 days) and from one time point (14 days) on malt extract reference medium, respectively, were analyzed by RNA-sequencing (Additional file 2: Figure S1b, c). According to the transcriptome analysis, 2 162 of the predicted *P. radiata* transcripts had significantly higher expression level (*p* < 0.05 and log2-fold change ≥1) and 1 820 had significantly lower (*p* < 0.05 and log2-fold change ≤−1) expression level at both or one of the time points on wood as compared with the malt extract cultivation. For specific transcripts, also more stringent *p* value and fold change threshold (*p* < 0.01 and log2-fold change ≥2) were applied to test for highly significant differences of gene expression between growth on wood and malt extract medium. The most highly upregulated transcripts on wood were recognized to encode candidate transporter proteins, hydrophobins, and candidate proteases together with various CAZymes (Table 2).

**Table 2:** Fifty most highly upregulated transcripts on 2 and/or 4 week time points of spruce wood cultivations of *P. radiata*

Gene id	Top 50 day 14	Top 50 day 28	Log2fc day 14	Log2fc day 28	Protein abundance (% of total MS intensity)					Function	Family
					7 days	14 days	21 days	28 days	42 days		
Minus.g6827	x		10.72	4.49	1.03	1.08	0.26	0.01	0.00	Lignin peroxidase (LiP2)	AA2
Minus.g11349	x	x	10.32	8.74	0.79	0.61	0.46	0.39	0.37	Alcohol oxidase	AA3
Plus.g9320	x	x	10.16	9.36	0.03	0.06	0.05	0.07	0.06	Lytic polysaccharide monooxygenase	AA9
Plus.g11539	x	x	9.96	8.43	0.02	0.07	0.10	0.14	0.07	Lytic polysaccharide monooxygenase	AA9
Plus.g2118	x	x	9.7	9.59						Oligopeptide transporter	
Minus.g10274	x	x	9.24	7.95						Lytic polysaccharide monooxygenase	AA9
Minus.g3073	x		8.71	1.72	0.91	0.32	0.10	0.00	0.00	Lignin peroxidase 1 (LiP1)	AA2
Plus.g1419	x	x	8.55	7.25	8.33	3.86	3.76	3.08	3.81	Manganese peroxidase (MnP1-long)	AA2
Plus.g1442	x	x	8.52	7.86	0.00	0.03	0.03	0.06	0.07	Acid protease, family A01A	
Minus.g10376	x	x	8.46	6.4						Hypothetical protein	
Minus.g3552	x	x	8.46	5.49	0.03	0.06	0.05	0.03	0.01	Lytic polysaccharide monooxygenase	AA9
Plus.g12321	x	x	8.43	7.48	0.01	0.01	0.03	0.01	0.01	Acetyl xylan esterase	CE1
Plus.g10527	x	x	8.07	8.37	0.00	0.05	0.14	0.06	0.04	Copper radical oxidase	AA5
Minus.g8138	x		8.07	5.16						Hypothetical protein	
Plus.g10872	x	x	7.62	6.5	0.29	0.39	0.23	0.45	0.68	Tripeptidyl peptidase, family S53	
Minus.g4805	x		7.62	4.5	0.15	0.15	0.11	0.06	0.02	Glutathione transferase	
Plus.g6944	x	x	7.61	7.75	0.00	0.00	0.01	0.02	0.03	Carboxypeptidase, family S10	
Minus.g2657	x	x	7.59	5.73						MFS general substrate transporter	
Minus.g3349	x	x	7.32	6.01						Carbohydrate-binding module family 1 protein	CBM1
Plus.g4342	x	x	7.2	7.32	0.20	0.32	0.22	0.23	0.10	Cellobiohydrolase	GH6
Minus.g4996	x	x	7.19	7.06						Sugar transporter	
Plus.g8760	x		7.19	3.71	0.15	0.22	0.10	0.01	0.00	s-adenosyl-l-methionine-dependent methyltransferase	
Minus.g11037	x		7.18	0.36	0.00	0.02	0.00	0.00	0.00	β-1,4-endoxylanase	GH10
Plus.g12778	x	x	7.12	7.36	0.06	0.14	0.28	0.70	1.29	GDSL-like lipase acylhydrolase	
Minus.g8600	x	x	7.08	6.33	0.00	0.01	0.02	0.15	0.16	Oxalate decarboxylase	
Minus.g3792	x	x	7.07	6.02						Hypothetical protein	
Plus.g3697	x		7.02	4.94	0.01	0.04	0.04	0.02	0.01	β-1,4-endoxylanase	GH11
Minus.g3846	x		6.96	5.43						Hypothetical protein	
Plus.g6610	x	x	6.89	5.87						Hexose transporter	
Minus.g10273	x	x	6.86	5.93						Lytic polysaccharide monooxygenase	AA9
Minus.g927	x	x	6.83	6.58	0.14	0.19	0.22	0.21	0.21	Tripeptidyl peptidase, family S53	
Plus.g6929	x	x	6.83	6.5						Hydrophobin	
Minus.g7795	x	x	6.73	6.38	0.05	0.07	0.11	0.08	0.05	Clavaminate synthase	
Minus.g3957	x	x	6.7	6.6	0.02	0.04	0.05	0.09	0.13	Acetylesterase	CE16
Plus.g11538	x		6.59	3.44	0.02	0.12	0.08	0.04	0.01	Lytic polysaccharide monooxygenase	AA9
Plus.g5095	x		6.58	2.97	0.00	0.04	0.03	0.01	0.01	NAD-binding oxidoreductase	
Minus.g2367	x		6.55	3.09	0.50	0.46	0.26	0.06	0.01	s-adenosyl-l-methionine-dependent methyltransferase	
Plus.g8273	x		6.5	5.14						Grp1/Fun34/YaaH domain transporter protein	
Plus.g13374	x		6.48	5.25	0.02	0.07	0.06	0.05	0.02	Lytic polysaccharide monooxygenase	AA9
Plus.g11441	x		6.46	3.65	1.65	2.24	1.93	1.23	1.09	NAD-dependent formate dehydrogenase	
Minus.g3726	x		6.36	4.61	0.00	0.01	0.02	0.01	0.01	Dihydrodipicolinate synthetase	
Plus.g917	x	x	6.33	6.17						Hydrophobin	
Plus.g3219	x	x	6.29	7.06	0.00	0.00	0.00	0.01	0.01	Carboxylesterase	
Plus.g4436	x		6.27	3.9						Hypothetical protein	
Minus.g9081	x		6.26	5.03	0.02	0.06	0.08	0.11	0.05	Exo-β-1,3/1,6-glucanase	GH131
Minus.g12191	x	x	6.23	5.7	0.07	0.11	0.07	0.09	0.03	β-1,4-endoxylanase	GH10
Plus.g4845	x		6.19	4.84	0.35	0.42	0.53	0.58	0.50	Short-chain dehydrogenase/reductase SDR	
Minus.g11036	x		6.18	4.12	0.03	0.03	0.02	0.02	0.01	β-1,4-endoxylanase	GH10
Minus.g9727	x	x	6.16	5.67						MFS general substrate transporter	
Minus.g9590	x	x	6.14	5.76	0.03	0.09	0.15	0.27	0.21	Peptidase, family G1	
Minus.g8467		x	6.13	5.61	0.04	0.05	0.08	0.07	0.09	Homoserine O-acetyltransferase	
Minus.g5595		x	6.07	5.55	0.16	0.54	0.34	0.18	0.04	Cellobiohydrolase	GH7
Minus.g10025		x	6.04	5.58	0.02	0.01	0.01	0.02	0.01	Acetylesterase	CE16
Minus.g9239		x	5.92	5.93	0.00	0.01	0.06	0.03	0.06	Unknown protein	
Plus.g4481		x	5.74	5.57						Cupredoxin domain protein	
Plus.g5796		x	5.7	6.17	0.02	0.01	0.02	0.01	0.02	Exo-β-1,3/1,6-glucanase	GH131
Plus.g8890		x	5.7	5.6						Hydrophobin	
Minus.g5364		x	5.57	5.65	0.00	0.02	0.06	0.11	0.27	α-glucuronidase	GH115
Minus.g7830		x	5.46	5.71	0.20	0.37	0.57	0.82	1.14	Alpha/beta-hydrolase	
Plus.g8163		x	5.4	6.73	0.01	0.05	0.13	0.39	0.53	Cellobiohydrolase	GH7
Plus.g4813		x	5.37	7.76	0.09	0.44	0.64	1.21	1.65	GMC oxidoreductase	AA3
Plus.g7451		x	5.36	5.51	0.09	0.22	0.16	0.18	0.09	β-1,4-endoglucanase	GH5_5
Minus.g3151		x	5.23	6.27	0.01	0.05	0.08	0.17	0.13	Mannosyl-oligosaccharide 1,2-α-mannosidase	GH47
Plus.g8155		x	5.22	6.2						WSC-domain-containing protein	
Plus.g6787		x	5.2	5.47						APC amino acid permease	
Minus.g4639		x	5.06	5.73						Glycoside hydrolase family 92 protein	GH92
Minus.g1646		x	4.95	5.72						Oligopeptide transporter	
Minus.g10423		x	4.07	5.49						Oligopeptide transporter	
Plus.g4812		x	3.24	5.79						Hypothetical protein	

Abundance values are given for proteins identified by peptide LC–MS/MS

### Wood-decay enzyme set of *P. radiata*

The emphasis of this study was on CAZyme encoding genes and especially on those identified as proteins and with known functions in degradation of plant cell wall components. The proportion of CAZymes, including GHs and CEs, was 7 % of the total proteome (Fig. 2). The auxiliary oxidoreductase activities covered 2 % of the total proteome with the AA2 family class-II peroxidases representing 32 % of this protein pool, that is 1 % of the total proteome. The proportion of GH proteins of the total proteome was relatively high (18 % of total proteome) at time point zero indicating active enzymatic carbohydrate utilization occurring by the fungal hyphae already in the malt extract inoculum cultivation. Overall distribution of proteins according to their CAZy families showed a large variety of functions (Additional file 4: Figure S3).

**Fig. 3:**
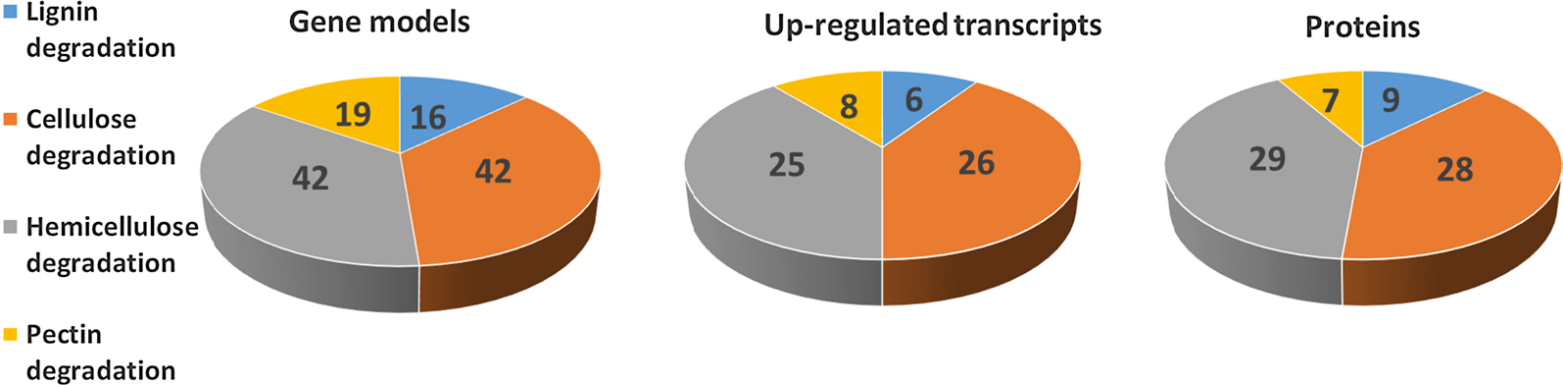
Comparison of functional distribution of plant cell wall degradation involved proteins in *P. radiata*, according to the annotated and translated gene models, upregulated transcripts in the wood transcriptome, and identified proteins from the wood proteome. Upregulated transcripts: transcripts having significantly (*p* < 0.05 and log2-fold change ≥1) higher level of expression at the specific time point on spruce wood relative to the malt extract cultivation

**Fig. 4:**
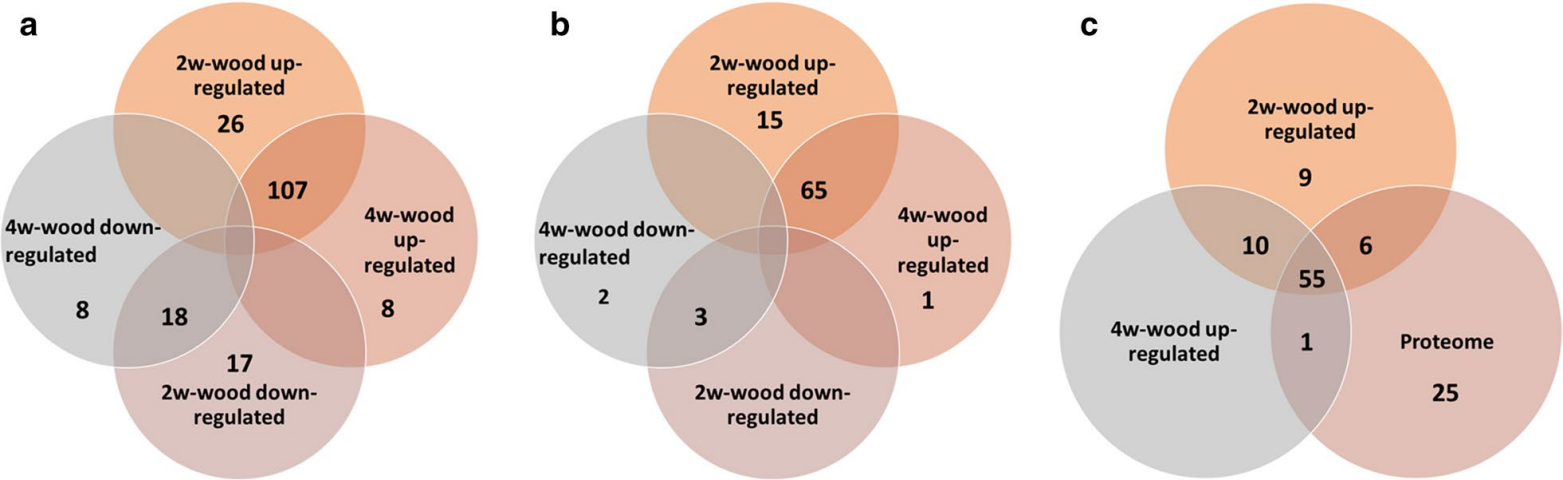
Distribution and regulation of CAZyme encoding transcripts at two time points on spruce wood presented in Venn diagrams. **a** Regulation of expression of all identified CAZy protein encoding transcripts. **b** Regulation of expression of plant cell wall degrading CAZy activities encoding transcripts. **c** Overlap of wood upregulated transcriptomes and total wood proteome of *P. radiata* for the plant cell wall degrading activities encoding transcripts and corresponding proteins. Upregulated and downregulated: transcripts having significantly (*p* < 0.05 and log2-fold change ≥1 or ≤−1) higher or lower level of expression at specific time points on spruce wood relative to malt extract cultivations. *2w* 2 week time point of cultivation; *4w* 4 week time point of cultivation

The number of upregulated transcripts and the number of detected proteins functionally annotated to correspond to plant cell wall degrading enzymes were compared, and the proportions were shown to be consistent (Fig. 3). Large proportion of the genes was upregulated and translated to proteins indicating active degradation of lignocellulose. In general, from the approximately 300 transcripts predicted to encode CAZymes belonging to different GH, CE, PL, and cellulose-binding module (CBM) families (excluding glycosyltransferases, GTs) together with AA9 lytic polysaccharide monooxygenases, AA2 class-II lignin-modifying peroxidases, and AA1 laccases, 141 were expressed on a significantly higher level on both or one of the transcriptome time points (14 and 28 days) on wood as compared with the 14-day malt extract cultivation. 107 of the transcripts were upregulated at both time points on wood (Fig. 4a). Majority (61 %) of these putatively encode activities against the plant cell wall (Fig. 4b). Moreover, majority of the transcripts upregulated at both time points on wood and coding for plant cell wall degrading activities were present in the proteome (85 %) (Fig. 4c).

**Fig. 5:**
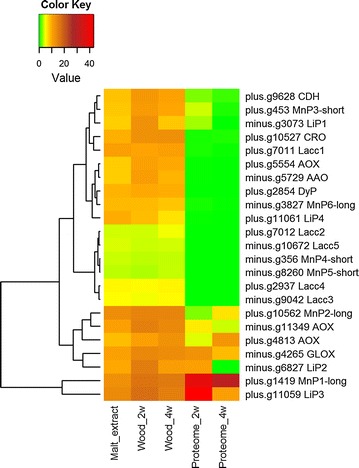
Heatmap presenting correspondence and clustering of expression of lignin-modifying and accessory oxidoreductase wood-decay CAZyme encoding transcripts and the corresponding protein production as identified in the proteome, at two time points during growth of *P. radiata* on spruce wood. *Columns* represent the culture conditions (Wood, Malt extract) and *rows* the transcripts and proteins (Proteome; total proteome on wood). *2w* 2 weeks; *4w* 4 weeks; growth on wood. *Color key* illustrates the rlog normalized expression values and the normalized abundance values for the transcripts and peptides, respectively. Gene model ids and annotated gene names are presented (right). *CDH* cellobiose dehydrogenase; *MnP* manganese peroxidase; *LiP* lignin peroxidase; *CRO* copper radical oxidase; *Lacc* laccase; *AOX* alcohol oxidase; *AAO* aryl-alcohol oxidase; *DyP* dye-decolorizing peroxidase; *GLOX* glyoxal oxidase

Upregulated transcripts involved in attack on lignin were encoding several class-II lignin modifying peroxidases, both LiPs and MnPs, upregulation being apparent at both time points on wood and being in agreement with the proteome data at the same time points (Fig. 5). The 20 most abundant CAZy proteins identified in the proteome samples included the most upregulated class-II peroxidases (Table 3). Moreover, transcripts for LiP2, LiP3, MnP1-long, and MnP3-short were highly significantly accumulated (*p* < 0.01, log2-fold change ≥2) during the wood cultivations.

**Table 3:** Twenty most abundant CAZymes in the proteome of *P. radiata*

Gene ID	Predicted function	Protein abundances						Average abundance on days 7–42	Transcripts up-regulated on wood
		Cultivation time (days)							
		0	7	14	21	28	42		
Plus.g1419	AA2: MnP1-Long	0.00	8.33	3.86	3.76	3.08	3.81	4.57	x
Plus.g11059	AA2: LiP3	0.00	5.37	4.32	2.58	1.10	0.70	2.81	x
Minus.g4265	AA5_1; glyoxal oxidase	0.00	2.32	1.25	0.75	0.88	2.10	1.46	x
Plus.g4813	AA3: GMC oxidoreductase	0.00	0.09	0.44	0.64	1.21	1.65	0.81	x
Plus.g10562	AA2: MnP2-Long	0.00	0.45	0.25	0.41	0.65	1.30	0.61	x
Minus.g11349	AA3: GMC oxidoreductase	0.00	0.79	0.61	0.46	0.39	0.37	0.52	x
Minus.g6827	AA2: LiP2	0.00	1.03	1.08	0.26	0.01	0.00	0.48	x
Minus.g7380	CE1	0.00	0.39	0.28	0.33	0.30	0.49	0.36	x
Plus.g453	AA2: MnP3-short	0.00	1.13	0.40	0.10	0.06	0.06	0.35	x
Minus.g2306	GH3	2.80	0.33	0.38	0.35	0.27	0.29	0.32	
Minus.g3073	AA2: LiP1	0.00	0.91	0.32	0.10	0.00	0.00	0.27	x
Minus.g5595	GH7	0.00	0.16	0.54	0.34	0.18	0.04	0.25	x
Plus.g8163	GH7	0.00	0.01	0.05	0.13	0.39	0.53	0.22	x
Minus.g6399	GH3	0.01	0.11	0.15	0.20	0.25	0.40	0.22	x
Plus.g4342	GH6	0.00	0.20	0.32	0.22	0.23	0.10	0.22	x
Plus.g637	CE16	0.00	0.18	0.13	0.15	0.27	0.33	0.21	x
Minus.g12190	GH10	0.00	0.24	0.23	0.21	0.22	0.08	0.19	x
Minus.g11677	GH28	0.76	0.53	0.16	0.04	0.02	0.01	0.15	
Plus.g7451	GH5	0.00	0.09	0.22	0.16	0.18	0.09	0.15	x
Plus.g9628	AA8-AA3_1: CDH	0.00	0.18	0.24	0.10	0.11	0.04	0.14	x
	Proportion (%) of total proteome	3.57	22.84	15.22	11.27	9.81	12.38	14.30	

Gene ID corresponds to annotated gene locus on the *P. radiata* genome assembly. Protein abundances are calculated based on mass spectrometric signal intensity values per each time point. Up-regulated transcripts refer to transcripts with significantly higher level of expression (*p* < 0.05 and log2-fold change ≥1) on wood as compared to the malt extract cultivations at both time points

*MnP* manganese peroxidase; *LiP* lignin peroxidase; *CE* carbohydrate esterase; *CDH* cellobiose dehydrogenase; *GH* glycoside hydrolase; *GLOX* glyoxal oxidase

The single dye-decolorizing peroxide encoding gene (DyP) which was annotated in the genome was transcriptionally up-regulated only at the earlier time point (14 days) on wood. Quite surprisingly, the respective protein was not detected in the proteome at any of the studied time points, from 0 to 42 days of wood colonization. Contrary to the DyP transcription response, a novel class-II peroxidase MnP6-long together with the previously cloned LiP4 was identified in the proteomes, although not observed being upregulated in the wood transcriptomes. In fact, both corresponding genes were downregulated at the later time point (28 days) on wood as compared with the malt extract reference transcriptome. On the contrary to the peroxidases, none of the five annotated laccase-encoding genes were regulated on wood, and Lacc1 protein was the only laccase enzyme present in the proteomes, taking into account all the time points analyzed. Noticeable is, however, that laccase activity was detected at every time point in the protein extracts of wood cultures (Additional file 3: Figure S2).

A large number of additional AA accessory oxidoreductases were found in the proteomes including alcohol oxidases, copper radical oxidases, one glyoxal oxidase (GLOX), GMC oxidoreductases, alcohol and aryl-alcohol dehydrogenases, one cellobiose dehydrogenase (CDH), and one benzoquinone reductase. Of this pool of H_2_O_2_ producing and other important oxidoreductive activities possessing enzymes, 11 were upregulated as transcripts at both time points on wood, with one alcohol oxidase gene transcript having the highest fold change. Transcripts for two alcohol oxidases, an aryl-alcohol oxidase, a GMC oxidoreductase, a GLOX, a copper radical oxidase, and a cellobiose dehydrogenase were highly significantly accumulated during the wood cultivations (Fig. 5).

**Fig. 6:**
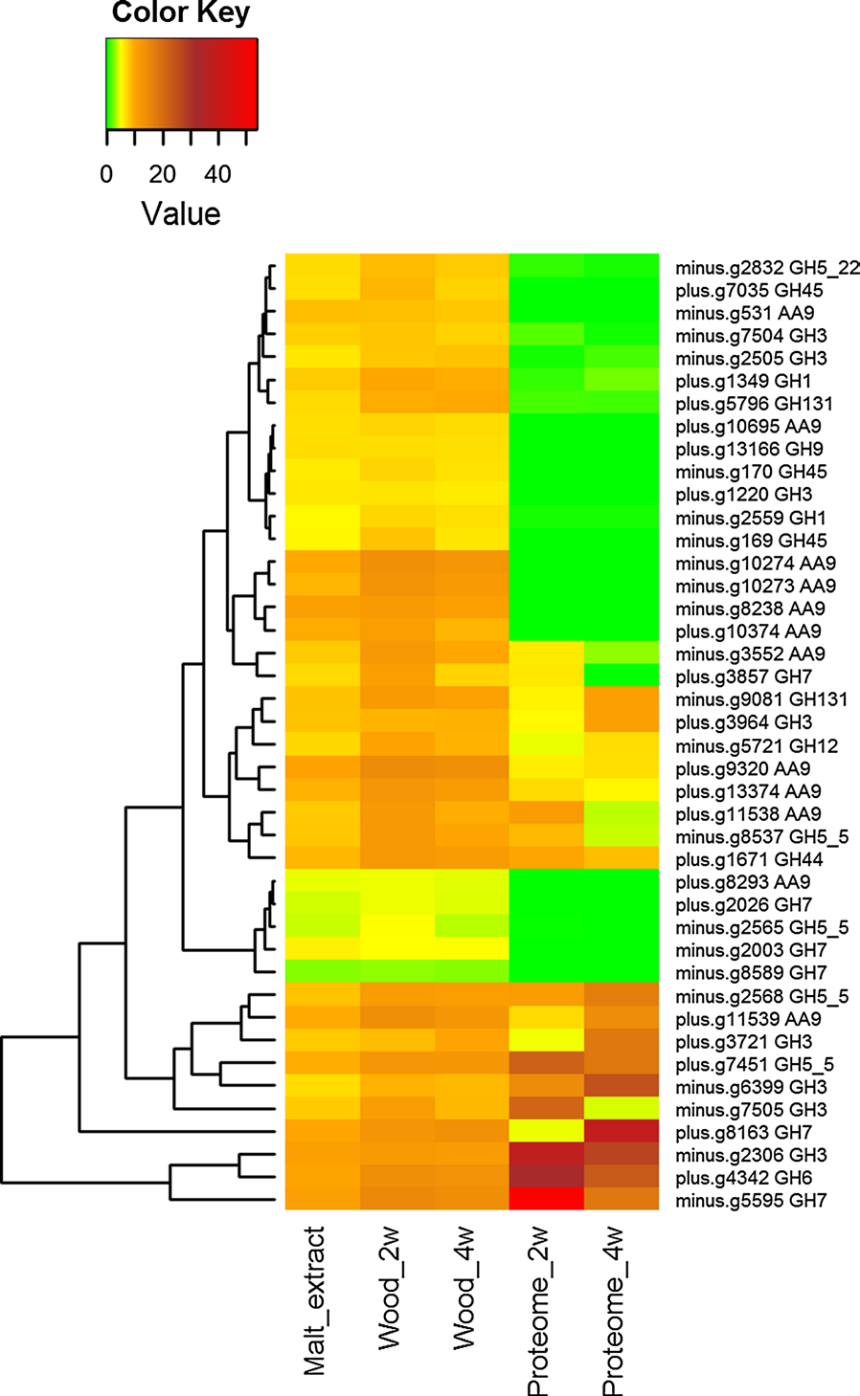
Heatmap presenting correspondence of expression of CAZy cellulose degrading enzyme encoding transcripts and respective protein production during growth of *P. radiata* on spruce wood. *Columns* represent the culture conditions and *rows* the transcripts/proteins. *Color key* shows the rlog normalized expression values and the normalized abundance values for the transcripts and peptides, respectively. Gene model ids and functional CAZy family annotations are presented (right). *GH* glycoside hydrolase; *AA* auxiliary activities. For other explanations, see Fig. 5

**Fig. 7:**
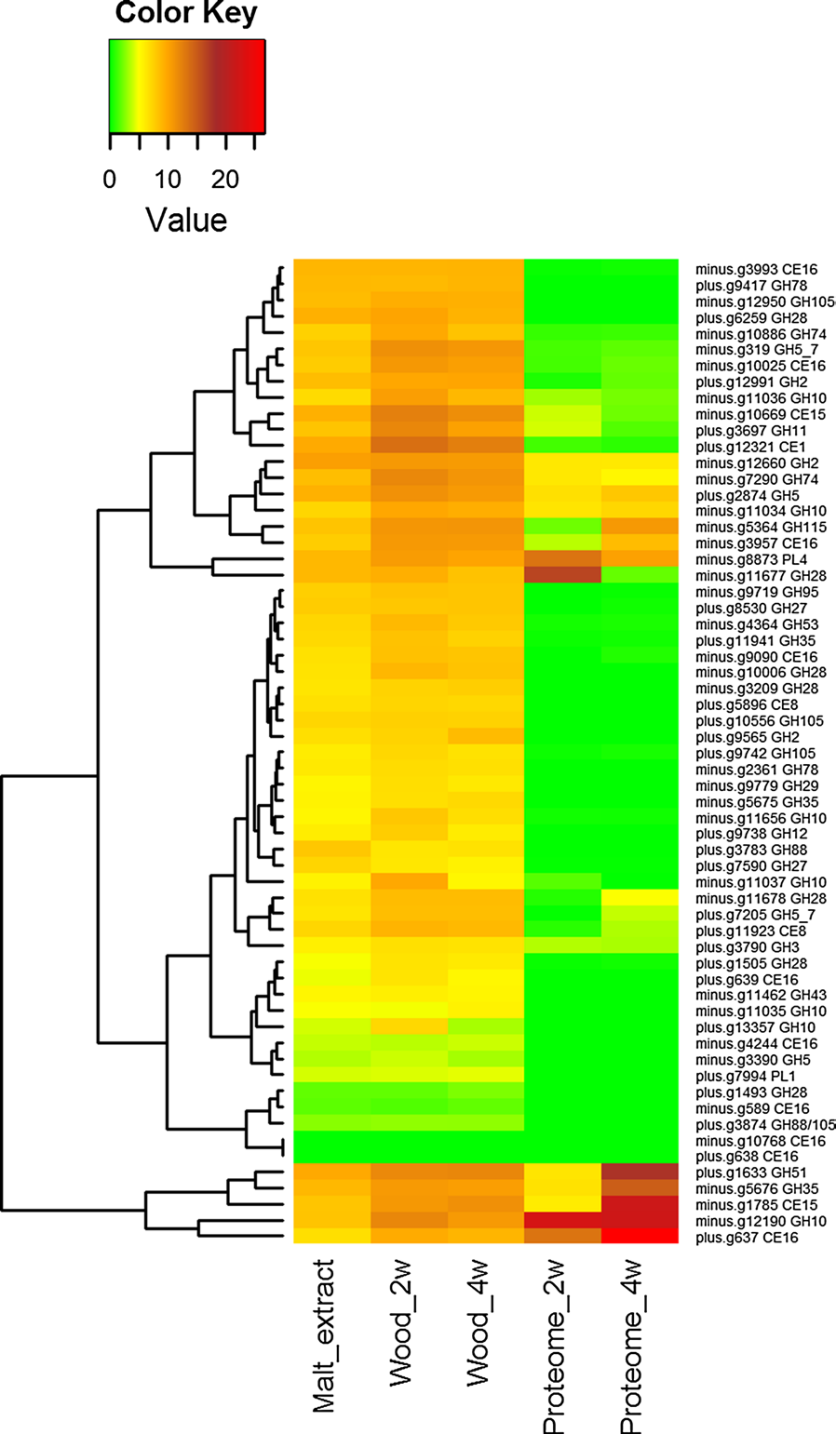
Heatmap presenting correspondence of expression of CAZy hemicellulose and pectin degrading enzyme encoding transcripts and respective protein production during growth of *P. radiata* on spruce wood. *Columns* represent the culture conditions and *rows* the transcripts/proteins. *Color key* shows the rlog normalized expression values and the normalized abundance values for the transcripts and peptides, respectively. Gene model ids and functional CAZy family annotations are presented (right). *CE* carbohydrate esterase; *GH* glycoside hydrolase; *PL* polysaccharide lyase. For other explanations, see Fig. 5

Transcripts from a number of different CAZy families, including activities against cellulose (Fig. 6) and hemicelluloses (Fig. 7), were upregulated independent of the time point on wood, and encode enzyme candidates, for example, AA9 LPMOs, cellobiohydrolases, β-1,4-glucosidases, β-xylosidases, β-1,4-endoglucanases, exo-β-1,3/1,6-glucanases, β-1,4-endomannanases, β-mannosidases, an acetyl xylan esterase, glucuronoyl esterases, acetylesterases, β-1,4-endoxylanases, an α-glucuronidase, an arabinofuranosidase, β-galactosidases, and xyloglucanases. Several of these genes, including seven genes encoding the AA9 LPMOs, were significantly differentially expressed on wood. These CAZy families were also present in the proteomes at least by one representative, and several of them were detected as being the most abundant CAZymes produced (Table 3).

A number of pectin-degrading CAZy genes, both lyases and hydrolases were expressed as transcripts on wood encoding for activities against polygalacturonan and rhamnogalacturonan, ester linkages (pectinesterase) and galactan. Many of these genes were upregulated at both time points on wood (Fig. 7). From these, three GH28 polygalacturonases, one CE8 pectinesterase, and the only PL4 rhamnogalacturonan lyase identified were highly significantly differentially expressed as transcripts. All of these—except one polygalacturonase enzyme—were also found as proteins in the proteome on wood. In addition, a candidate GH43 galactan 1,3-β-D-galactosidase possibly involved in degradation of arabinogalactan and transcripts encoding CBM1 modules and one cytochrome b562 iron reductase with a CBM1 domain were upregulated at both time points on wood (also when more stringent p-value and fold change were applied). From these, only the GH43 protein was present in the proteome.

In total, a portion (7.3–8.6 %) of the proteins identified in the total proteome on wood was classified as proteins with unknown function. Although none of these were among the 20 most abundant proteins produced on wood (Additional file 1: Table S1), they may include a few candidates that are important for fungal wood decay, thus presenting interesting targets for future studies. One of these is the transcriptionally highly upregulated *P. radiata* gene model minus.g9239 with unknown function but with the corresponding peptides discovered in the wood proteome (Table 2).

### Dynamics of plant cell wall degrading enzymes

**Fig. 8:**
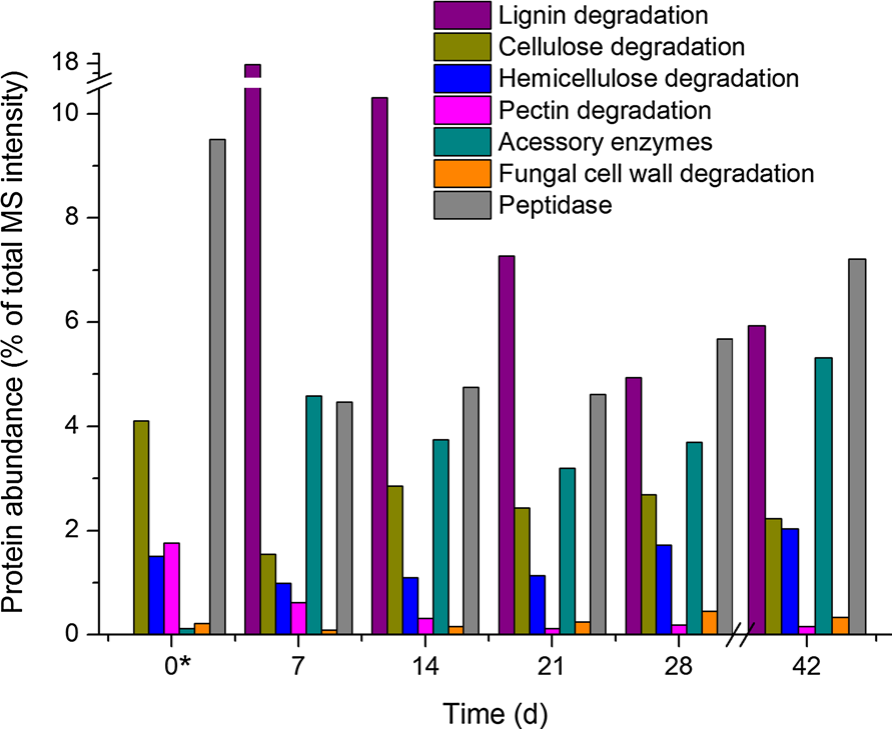
Net abundances for CAZy plant and fungal cell wall degrading and accessory enzymes of *P. radiata* at each week and studied time point on spruce wood cultivations. *The total proteome at time point zero was the malt extract inoculum culture mycelium which contained approximately 1/10 of the number of identified proteins detected at the other time points (Table 1)

When time-dependency of protein expression was studied, it was noticed that despite the fact that protein numbers within each functional category were somewhat constant during the cultivation (Fig. 2), differences in relative abundances of the proteins were observed (Fig. 8). When fungal proteins predicted to be important for wood decay were analyzed at every time point, the largest expansion seemingly occurred in the pool of identified proteins (of the overall protein production) during the first cultivation week. The abundances of the lignin-attack associated proteins peaked at growth day 7, and as the cultivation time advanced, abundances of these proteins started to decline. The only exception was the class-II peroxidase MnP2-long, the abundance of which started to increase again on cultivation day 17 (Additional file 5: Table S2).

Especially, the LiP abundance was decreasing, whereas MnP proteins were either constantly produced or demonstrating a less dramatic decline in protein abundances during the cultivation. This phenomenon was also observed in the transcriptome with the expression of especially LiP transcripts decreasing after 14 days on spruce wood. The MnP enzyme activities were constant on wood and detected also from the last time point (Additional file 3: Figure S2). Accessory oxidoreductase enzymes demonstrated likewise constant protein production during the cultivation. The abundancy of one GLOX protein (encoded by the gene model minus.g4265) of family AA5_1 declined between days 21 and 28 then increasing again until the end of the cultivation on wood (day 42). In addition, various dehydrogenases belonging to GMC oxidoreductases were produced with low protein abundances.

Cellulose degrading enzymes peaked in the proteomes on wood on cultivation day 14 (Fig. 8). Of the CAZy family GH7 cellobiohydrolases, relative abundance of one protein (encoded by the gene model minus.g5595) was clearly decreasing during the cultivation whereas the abundance of another GH7 protein (encoded by the gene plus.g8163) increased. The transcriptome data indicated corresponding differences in gene expression indicating that these two GH7 cellobiohydrolase encoding genes are under differential regulation. Relatively low cellobiohydrolase activities were, however, measured after the third week of cultivation on wood (Additional file 3: Figure S2). The highest abundance of β-glucosidases was detected for proteins of CAZy family GH3 while family GH1 proteins remained low in abundance. β-glucosidase activities in turn were measured throughout the cultivation (Additional file 3: Figure S2). Relative abundances of hemicellulose degrading enzymes increased in the proteome on wood during the active growth of *P. radiata*, and xylanase activity was detected at time points 7, 28 and 42 (Fig. 8; Additional file 3: Figure S2). Production of CAZy family GH35 β-1,4-galactosidase, a GH51 α-arabinofuranosidase and a GH115 α-glucuronidase proteins increased during the cultivation. The total abundance of pectin degrading proteins was quite low during the cultivation on wood.

To follow the dynamics of fungal cell wall degradation and hyphal autolysis, abundances of twelve chitinase and β-glucanase proteins belonging to GH families 5, 13, 16, 18, 20, 55, 72, and 92 were followed. Abundances of these proteins were increasing up to day 28 of the wood cultivation (Fig. 8). It is of interest that the abundance of peptidases (protease activity proteins) followed a similar pattern although the protein numbers (number of individual gene products) and their abundances were higher. Identified peptidases represented 27 different MEROPS peptidase families. Majority of the peptidases were assigned to A01A and T01A subfamilies. The top three most abundant peptidases included a family M28 metalloprotease, an A01 aspartyl peptidase and an S53 tripeptidyl peptidase.

### Decay of spruce wood by *P. radiata*

**Fig. 9:**
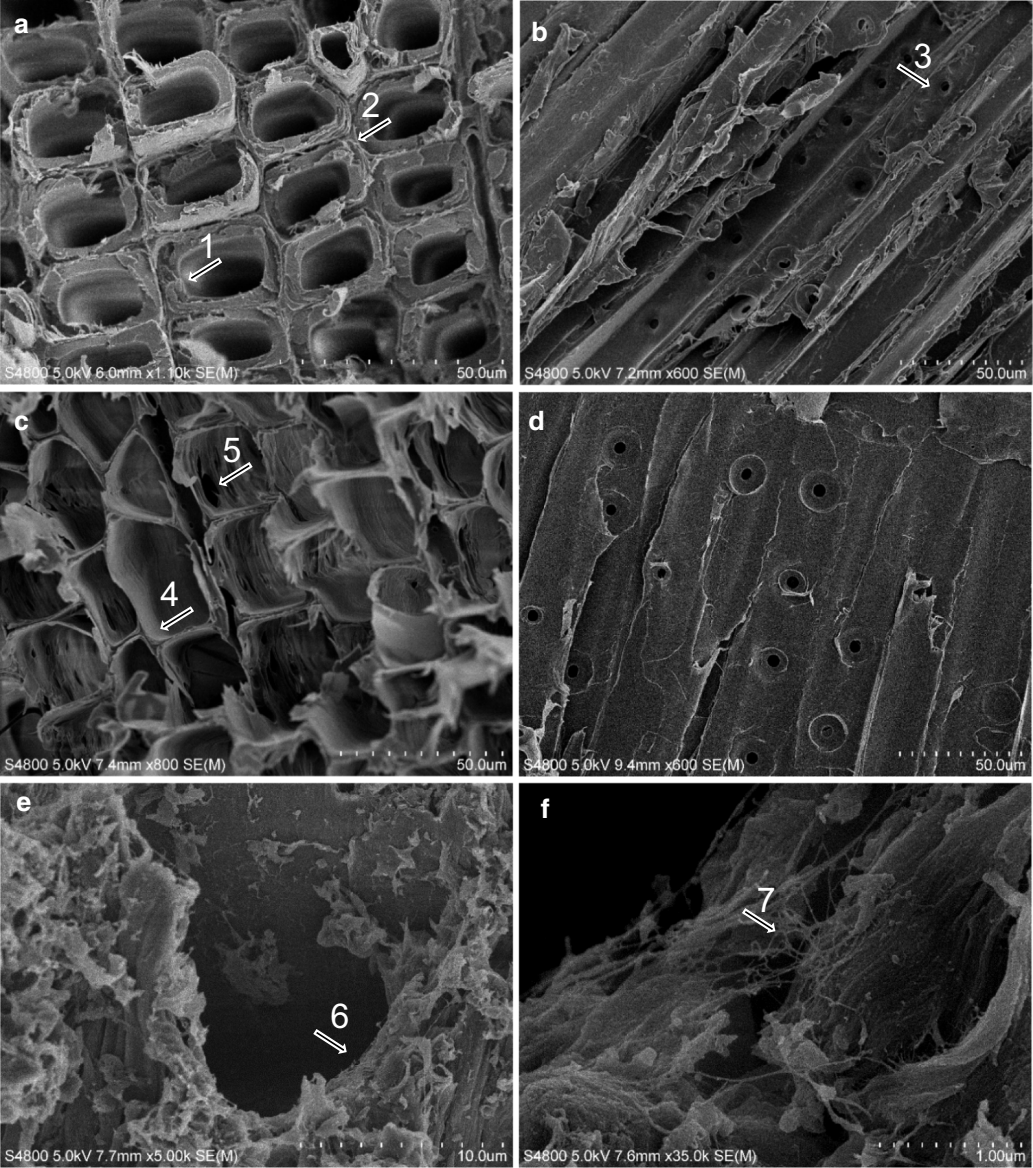
Field emission scanning electron microscope images of Norway spruce xylem after 42 days of solid-state growth and decay of spruce sticks by *P. radiata*. Transverse (**a**) and longitudinal (**b**) sections of non-inoculated wood. The *arrows* indicate *1* intact spruce wood cell secondary wall, *2* middle lamella, *3* bordered pits at the tracheid walls. **c** Transverse section of spruce xylem after decay by *P. radiata* demonstrating enlargement of the tracheid lumen volume and apparent thinning of secondary cell wall. The *arrows* indicate *4* thinned remains of secondary cell wall, *5* enlarged bordered pit. Notice detachment of the tracheids due to erosion of primary cell walls and middle lamellae. **d** Vertical section of fungal decomposed spruce xylem. Tracheids are flattened and detached due to secondary cell wall thinning and erosion of middle lamellae. **e**, **f** Transverse sections of fungal colonization of xylem observed with higher magnification (5000 and 35,000x) demonstrating the hyphae *6*, *7* as threads inside the tracheids. Notice glucan deposits (**e**) and release of tracheid cell wall layers. *Scale bars* are in (**a**, **b**) 50 µm, (**e**) 10 µm and (**f**) 1 µm

Vertical and transverse sections of spruce wood samples were observed by field-emission scanning electron microscopy. Intact xylem and wood cell wall ultrastructure with tracheid bordered pits were observable in non-inoculated spruce wood (Fig. 9a, b). After 42 days of growth, *P. radiata* hyphae were visible particularly inside the tracheids (in tracheid lumen) and attached to secondary cell walls (Fig. 9e, f). In fungal colonized wood samples, a few enlarged bordered pits and thinning of tracheid cell walls were noticeable (Fig. 9c, d).

Supporting results were obtained by lignin analyses at first determining the gravimetric Klason lignin content and by more accurate analytical methods (Table 4). Klason lignin content and total yield of aromatic compounds detected by pyrolysis–GC/MS analyses were relatively constant in the fungal decayed wood samples compared to non-inoculated wood but some changes were, however, observed in lignin structure and polymerization stage. By pyrolysis–GC/MS analyses, in total, 20 compounds originating from lignin substructure units were tentatively identified according to MS spectra and reference compounds (Table 4). Increase in the abundance of monomeric phenolic compounds such as methylated phenols and guaiacol was probably caused by attack on lignin moieties resulting with decrease in the molecular size of polymeric lignin and release of oligomeric and monomeric substructures during fungal decay. This was also observed as an increase in the content of acid soluble lignin. The ratio of phenylmethane and phenylethane units to phenylpropane units (Ph-C1,C2/Ph-C3 ratio) increased after fungal cultivation indicating that a part of the side chain linkages of lignin substructures were cleaved. Lignin oxidative and degradative activity was also observed as increment of the amount of phenolic compounds, such as vanillin and coniferyl aldehyde (Table 4).

**Table 4:** Lignin composition analysis of Norway spruce wood after 6 weeks of colonization by *P. radiata*

RT	Peak name	Spruce wood (%)	After 6 weeks of fungal growth (%)	Change (%)
		Mean	Mean	
7.126	Phenol (hydroxybenzene)	0.29 ± 0.08	0.50 ± 0.09	+72
8.401	2-methylphenol	0.11 ± 0.03	0.16 ± 0.03	+45
8.753	4-methylphenol, (4-methylguaiacol)	0.25 ± 0.06	0.33 ± 0.05	+32
9.01	2-methoxyphenol, (Guaiacol)	3.33 ± 0.24	4.69 ± 0.30	+41
10.437	2-methoxy-3-methylphenol	0.76 ± 1.22	1.24 ± 0.99	+63
10.64	2-methoxy-4-methylphenol	3.19 ± 0.47	2.44 ± 0.47	−24
11.89	2-methoxy-4-ethylphenol, (ethylguaiacol)	0.83 ± 0.10	0.73 ± 0.08	−18
12.428	4-vinylguaiacol	3.17 ± 0.29	3.22 ± 0.39	+2
12.994	Eugenol, 4-allylguaiacol	0.57 ± 0.05	0.45 ± 0.02	−21
13.129	2-methoxy-4-propylphenol, (4-propylguaiacol)	0.31 ± 0.03	0.26 ± 0.04	−16
13.626	Vanillin	1.27 ± 0.56	1.45 ± 0.71	+14
13.692	2-methoxy-4-(1-propenyl)phenol, (isoeugenol, *cis*)	0.27 ± 0.02	0.23 ± 0.02	−15
14.274	2-methoxy-4-(1-propenyl)phenol, (isoeugenol, *trans*)	1.78 ± 0.18	1.47 ± 0.25	−17
14.355	1-(4-hydroxy-3-methoxyphenyl)ethanone, (guaiacylacetone)	1.06 ± 0.65	0.86 ± 0.65	−19
14.733	1-(4-hydroxy-3-methoxyphenyl)ethanone (acetovanillone)	0.98 ± 0.46	0.54 ± 0.47	−45
15.201	Phenol, 2-methoxy-4-propan-1-ol, (dihydroconiferyl alcohol)	0.81 ± 0.54	0.35 ± 0.27	−57
15.749	Coniferyl alcohol, *cis*	0.47 ± 0.25	0.24 ± 0.17	−49
15.867	1-(4-hydroxy-3-methoxyphenyl)propanone, (propiovanillone)	0.58 ± 0.27	0.35 ± 0.30	−40
16.657	Coniferyl alcohol, *trans*	0.60 ± 0.46	0.35 ± 0.46	−42
17.673	Coniferyl aldehyde	0.51 ± 0.35	0.52 ± 0.59	+2
Pyrolysis lignin (area aromatics/total)		21.3 ± 0.7	20.4 ± 2.0	−4
Gravimetric (Klason) lignin		25.8 ± 0.33	26.1 ± 0.51	+1
Acid soluble lignin		0.30 ± 0.04	0.68 ± 0.23	+127
Pch-C1,2/Pch-C3 ratio^a^		0.03	0.08	+167

Relative peak areas (%) of lignin-derived pyrolysis product compounds identified with pyrolysis–GC/MS. Gravimetric and acid soluble lignin contents are also shown. The values are calculated from two biological replicates where 3–4 technical replicates were taken for pyrolysis samples, and from three biological replicates each of which with two technical replicate samples for Klason lignin determination

^a^Ratio of phenylmethane and phenylethane to phenylpropane type compounds

## Discussion

White-rot fungal secretomes on various lignocelluloses have been investigated largely concentrating on the model white-rot fungus, Agaricomycetes Polyporales species *Phanerochaete chrysosporium* [16, 33, 34]. Similar studies on phlebioid clade fungi, including *Phanerochaete carnosa* [35], *Phlebiopsis gigantea* [36] and *Irpex lacteus* [37], as well as on the other white-rot Polyporales species *Ganoderma lucidum* [38], *Ceriporiopsis subvermispora* [39, 40], *Pycnoporus cinnabarinus* [41], *Pycnoporus coccineus* [42], and *Trametes trogii* [43], were conducted recently. In addition, lignocellulose-decay proteomics and transcriptomics of the order Agaricales white-rot species *Pleurotus ostreatus* have been elucidated [44, 45]. With these data and the accumulating genomic knowledge on fungi, however, it is evident that there are some differences in gene numbers but larger variations in gene expression and CAZy protein production between the white-rot fungal species. Moreover, these differences seem to be more fungal species and isolate dependent than influenced by, e.g., the type of wood and lignocellulose used as growth substrate [35, 42, 45].

Regarding this, we aimed at characterisation of the CAZy proteins in the total proteome on wood of an efficient lignin-degrading white rot species, *P. radiata*, isolate 79, not fully investigated by omics approach before but with a recently sequenced and annotated genome available. This allowed us to perform an extensive and deep time point study on the proteome and transcriptome while the fungus is colonizing wood. Moreover, the time point study allowed us to observe dynamic changes in the abundances of *P. radiata* proteins expressed on wood, and to gain insight into regulation of CAZy gene expression by transcriptome analyses. Our results confirm that the wood-decaying enzyme repertoire of *P. radiata* is functional and composed of a variety of CAZy families including the auxiliary oxidoreductases, a set of lignin-modifying peroxidases accompanied by H_2_O_2_ producing enzymes and LPMOs, and a selection of hydrolases, esterases and lyases, all needed for complete degradation of the polymeric lignocellulose components.

The wood-decay machinery of *P. radiata* is typical for a traditional white-rot fungus (including various class-II lignin-modifying peroxidases, laccase, CDH, GMC oxidoreductases, GLOXs, LPMOs, cellulases of GH5, GH6, GH7 families) [1–3, 46]. However, some unique features like high expression of several LiPs and both long- and short-MnPs but low expression of laccase differentiate *P. radiata* from many other white-rot species of Polyporales. Strong expression of multiple lignin-modifying LiP and MnP class-II peroxidases, together with H_2_O_2_ producing oxidoreductases (GLOX, GMCs) and GH6 and GH7 cellobiohydrolases is more similar to the spectrum of CAZymes expressed by *P. chrysosporium* [7, 47]. In another phlebioid clade species, *Phlebiopsis gigantea*, ten genes codifying for class-II peroxidases were identified, but none of these were detected as secreted proteins in pine wood containing cultures [36]. Instead, one DyP (dye-decolorizing peroxidase) was identified in the *P. gigantea* secretome [36]. In our study, the only DyP encoding gene annotated in *P. radiata* genome was detected in the wood transcriptome but not identified in the proteome. These findings pinpoint that each phlebioid species expresses a unique array of CAZy and oxidoreductase enzymes and have an exquisite strategy for colonization and decay of wood.

Preliminary annotation of the *P. radiata* genome assembly revealed ten genes for class-II lignin-modifying peroxidases. Of the four LiPs, the well-characterized LiP3 enzyme [30] was the most abundant protein in the proteome with high expression level on wood, although transcriptionally not as strongly upregulated as LiP2 and LiP1 encoding genes. Three of the *P. radiata* LiP encoding genes (LiP1, LiP3, and LiP4) have been cloned and characterized with the respective transcripts shown to be expressed by the fungus on milled spruce and alder wood supplemented cultures [24]. Furthermore, LiP1 protein was previously detected in Norway spruce shavings containing bioreactor cultivations of *P. radiata* [48].

Similar to LiP production, variations were observed in this study for expression of the six MnP encoding genes as transcripts and proteins on spruce wood. A novel *P. radiata* long-MnP1 encoding gene was observed as highly upregulated on spruce wood and correspondingly produced in high protein amounts throughout the cultivation. In contrast, of the two previously cloned and characterized *P. radiata* MnPs [25], expression of the long-MnP2 showed an increasing tendency, while the short-MnP3 demonstrated protein decline during the six-week surveillance on wood. This indicates that the multiple class-II lignin-modifying peroxidase encoding genes are differentially regulated in fungi, with some genes being quickly responsive to the wood substrate while transcription of the others may be more subjected to time and fungal growth-dependent regulation.

Previously on solid-state wheat straw lignocellulose cultures of *P. radiata* 79, LiP2 enzyme was detected as one of the major extracellular proteins produced by the fungus together with a few GLOXs [49]. Moreover, LiP and GLOX activities were observed to decline between cultivation days 14 and 28 [49], accordingly as is observed in our present study for the several LiPs indicating a decline in protein abundances in the later stages of the six-week cultivation on spruce wood. Taken together, our results confirm the previous observations for *P. radiata* and indicate synergistic action of GLOXs and LiPs when the fungus is growing on lignocelluloses likewise is reported for *P. chrysosporium* [7, 50, 51].

Together with the CRO glyoxal oxidases of the CAZy family AA5, GMC aryl-alcohol oxidases from CAZy family AA3 may also supply extracellular hydrogen peroxide for the fungal wood decay and class-II peroxidases, and AA3 oxidoreductases may also act as coupled activities to aryl-alcohol dehydrogenases [52]. Both AA3 and AA5 proteins were detected on *P. radiata* spruce wood cultivations together with various other alcohol oxidases and dehydrogenases possibly working as accessory oxidoreductive enzymes and having a role in enhancing attack on wood lignin. Expression of the latter transcripts may be a result of active intracellular modification of lignin metabolites as is reported for *P. chrysosporium* [47].

Laccase activities were measured in our spruce wood cultivation and in previous studies on wood-containing cultures of *P. radiata* [18, 53]. Five laccase encoding genes are recognized in the *P. radiata* genome. However, only one laccase protein, that is the well characterized *P. radiata* Lacc1 [26, 27], was identified in constant but low amounts in the wood proteomes at the time points studied. Lacc1 protein has been repeatedly detected in liquid media and solid lignocellulose cultures of the fungus [49, 53–56] emphasizing its main role in *P. radiata* secretome regardless of the growth substrate and carbon source. The transcriptome revealed that several laccase encoding genes were expressed on wood although *lacc1* gene had clearly the highest transcript abundances. None of the laccase encoding genes, however, was significantly upregulated on wood, which indicates constant expression in particular for *lacc1*.

The constant expression of laccase encoding genes with only one secreted protein may reflect the evolutionary relationship of *P. radiata* and systematic placement in the phlebioid clade of Polyporales. Phlebioid clade includes fungal species completely lacking laccase encoding genes in their genomes, such as *Phanerochaete chrysosporium, Phanerochaete carnosa,* and *Phlebiopsis gigantea* [36, 57, 58]. On the contrary, lignocellulose secretomes of more far-related white-rot fungal species from other systematic clades and orders of Agaricomycetes demonstrate a wider array of laccase proteins, e.g., in *C. subvermispora* (three detected laccases on lignocellulose), *Pleurotus eryngii* (four detected laccases), and *Pleurotus ostreatus* (four detected laccases) [39, 43, 45, 59]. In this respect, the phlebioid white-rot fungi demonstrate their own type of (less or non-laccase dependent) strategies of wood-decay.

In addition to lignin attack, cellulose degradation by white-rot fungi occurs via the combination of several divergent protein families, that is by cellobiohydrolases, LPMOs and CDH [60]. LPMOs from CAZy family AA9 are important in cellulose and hemicellulose degradation [61–63], and they may utilize various electron donors including CDH, haem proteins fused to cellulose-binding module (cytochrome b562-CBM1), or lignocellulose-derived and fungal produced di-phenols [63–65]. In addition, the GMC oxidoreductases, such as glucose oxidase and pyranose dehydrogenase, may participate in the redox system [63]. Transcripts of *P. radiata* encoding LPMOs and the assisting activities were identified on wood. Seven of the twelve annotated LPMO encoding genes were highly expressed and significantly upregulated on wood, and five of these were accordingly identified as peptides in the proteome. Moreover, supporting the oxidative and electron transfer oxidoreductive protein attack on wood generated by fungal produced LPMOs, CDH protein— having variable suggested catalytic roles [66]—was one of the most abundant CAZymes identified in the *P. radiata* wood proteome and was accordingly upregulated in the transcriptome.

Additional enzymes important in fungal carbohydrate metabolism and connected to wood-decay are aldose-1-epimerases (ALE) [67] that generate cellobiose β-anomers, which in turn are reducing substrates of CDH enzymes. Three putative ALEs were found in the wood proteome of *P. radiata*. One *ale* (gene model minus.g8594) was upregulated in the wood transcriptome. ALE encoding genes are apparently general in wood-decay fungi and identified in white-rot and brown-rot Polyporales genomes [67]. Corresponding peptides were supportively detected in secretomes of *Phanerochaete chrysosporium* but not in *Phlebiopsis gigantea* [13, 33, 63].

To study the effects of hyphal growth on wood on the fungal cell wall reorganization and degradation, the abundances of fungal cell wall degrading proteins during the cultivation were followed together with peptidases. Abundances of these proteins increased over time, thus indicating the need of *P. radiata* to recycle essential nutrients and reorganize the fungal hyphae and cell wall upon growth and colonization of wood. It has been suggested that protease expression is connected to nitrogen acquisition from fungal produced and lignin-linked proteins in the nitrogen-limited wood environment [68–70]. Several other nitrogen metabolism associated transcripts were also upregulated in *P. radiata* on wood including oligopeptide transporters similar to as is observed in *P. chrysosporium* [7], although the corresponding proteins were absent from our proteome samples.

In addition to nutrient cycling and degradation of intracellular proteins, fungal proteases have been suggested to be important for β-1,4-endoglucanase activation [71] and cleavage of the CDH flavin-containing protein domain [72]. Proteases have previously been connected to the decline of extracellular LiP activities in fungal cultures [73, 74]. In this study, the initially very high and upregulated LiP abundances were slowly decreasing as protease abundances were increasing in the *P. radiata* wood proteome, thus indicating that some part of the highly secreted and produced enzymes may be degraded by the fungus’ own proteases to recycle the otherwise scarce organic nitrogen pool in the high C/N ratio wood environment. Overall, high peptidase abundances in secretomes of plant biomass-degrading saprotrophic Basidiomycota have been observed [75].

Differences between the abundances of expressed transcripts and detected proteins, and their relation to enzyme activity values at specific time points of the wood cultivation reflect differential and perhaps time-dependent regulation of fungal genes. Alternating processes of gene regulation occurring during (transcriptional regulation) and after transcription (post-transcriptional regulation) may affect the outcome of transcriptomics and proteomics studies. Distinct isoenzyme proteins that are products of individual genes may in turn be active only at certain phase of wood degradation or under specific environmental conditions such as ambient pH [76, 77]. Thus, gene expression analyses, protein detection, and enzymatic activity measurements are not always directly comparable. Moreover, low-molecular-weight proteins, proteins without trypsin cleavage sites or those left intact and attached to the wood matrix, as well as quickly degraded proteins are usually underestimated or lost entirely by peptide LC–MS/MS [39]. However, the clear correlation in time-dependent (higher at the earlier time points) production of proteins with transcript level expression of *P. radiata* genes encoding the various class-II peroxidases indicates strong transcriptional activation of the genes when the fungus is in contact with wood, a natural lignocellulose substrate for hyphal colonization and growth.

After 42 days of wood colonization, it appears that *P. radiata* causes simultaneous decay of spruce xylem components proceeding from the wood cell empty lumen side to secondary cell walls towards primary cell wall and tracheid middle lamellae. This is seen as evident thinning and erosion of the secondary cell walls, together with some erosion of middle lamellae and release of the tracheids. Similar wood-decay pattern is observed for other white-rot Polyporales species on gymnosperm wood [78]. Clear indication of degradation of lignin moieties and release of phenolic compounds during the cultivation period (42 days) was demonstrated by pyrolysis–GC/MS although the gravimetric Klason total lignin content (in relation to wood dry weight) demonstrated a slight increase. This may be explained by the apparent simultaneous degradation of wood cell wall cellulose and other polysaccharides at this stage, thus affecting the ratio of lignin content versus content of wood carbohydrates. Coinciding results were obtained in *P. chrysosporium* cultivations on softwood after 21 days [79].

In a previous study, up to 22 % decrease in Klason lignin content was obtained with the same fungus *P. radiata* 79 after 70 days of cultivation on Norway spruce wood chips [22]. In our study, spruce wood lignin was attacked and degraded during 42 days by *P. radiata* to some extent. Pyrolysis–GC/MS analysis demonstrated decrease in the amount of spruce wood phenylpropane units with concomitant increase in the number of smaller fragmented products from these lignin units, suggesting that especially the upregulated lignin peroxidases were actively attacking and converting the predominant non-phenolic structures of the spruce wood coniferous lignin. Lignin peroxidase LiP3 of *P. radiata* is an efficient oxidizer and degrader of non-phenolic lignin β-O-4 dimeric structures [30, 55]. *P. radiata* short-MnP3 is active against phenolic lignin compounds [29] and in decomposition of pine wood lignin [28]. Increment of phenolic units after fungal growth on lignocellulose was likewise observed in *P. chrysosporium* on pre-treated biomass [80]. Although chemical and structural evidence of spruce wood lignin decay was obtained with *P. radiata*, it appears that a somewhat longer cultivation time (than 6 weeks) is needed to observe a decrease in total lignin content as well as more dramatic wood ultrastructural changes leading to complete tracheid (wood fiber) separation by degradation of middle lamellae.

## Conclusions

It appears that *P. radiata* initiates a strong oxidoreductase and lignin-attacking enzyme expression in contact with wood, which is seen in high transcription upregulation and protein production already during the first weeks upon wood colonization. After this, together with the lignin-attacking and auxiliary oxidoreductases, an array of cellulose and hemicellulose acting GH and CE enzymes is produced, with some changes in protein abundances in the course of the 6 week surveillance and growth on wood. Thus, after the initial lignin-attacking reactivity, at the second stage of wood decay, the *P. radiata* CAZyme repertoire is more targeted against the wood polysaccharides. The change of strategy is most likely to support supply of readily metabolized carbohydrates (sugars) for energy and biosynthetic metabolic processes. After 42 days of wood colonization, the fungal decay is seen as erosion of the wood cell walls with some release of the tracheids, with evident attack on the lignin subunits leading to release of phenolic and lignin-derived fragmented compounds.

In general, transcriptome analysis supported the proteomic results, thus confirming that majority of the identified CAZy encoding transcripts which were upregulated on wood were also present in the proteome. The transcriptome data indicated a pronounced role especially for a few lignin peroxidases (LiP2, LiP3) and manganese peroxidases (MnP1-long, MnP2-long, MnP3-short), various hydrogen peroxidase generating accessory enzymes (GLOX, AA3 AOX), CE acetyl xylan esterase, and lytic polysaccharide monooxygenases (LPMOs), when *P. radiata* is actively colonizing and degrading coniferous spruce wood. In addition, various CAZymes against cellulose, and both glucomannan and xylan type of hemicellulose together with constant expression of pectin-degrading enzymes were detected, all adding up to the impressive repertoire of lignocellulose-attacking enzymes expressed by *P. radiata* upon colonization of spruce wood.

## Methods

### Fungal strain

*Phlebia radiata* Fr. (isolate 79, FBCC0043), previously collected in South Finland and isolated from decayed gray alder (*Alnus incana*) wood, was obtained from the HAMBI Fungal Biotechnology Culture Collection (HAMBI-FBCC, fbcc@helsinki.fi) of the University of Helsinki, and cultivated and maintained on 2 % (w/v) malt extract agar plates at 25 °C and in the dark.

### Cultivation conditions

For fungal inoculum, *P. radiata* was cultivated in 75 ml liquid 2 % (w/v) malt extract broth, which was inoculated with four mycelium-covered plugs (7 mm in diameter) from malt agar plates, and incubated for 7 days at 25 °C. The inoculum culture was homogenized using Waring blender to initiate either 100 ml portions of liquid 2 % (w/v) malt extract medium or spruce wood cultures by using 2 ml of the homogenized fungal mycelium. The solid wood cultivations contained 2 g (dry weight) of autoclaved Norway spruce (*Picea abies*) wood sticks (dimensions about 25 × 3 × 2 mm) on a 1 % (w/v) water agar with a total moisture content of 60 %. All cultivations were incubated under stationary conditions at 25 °C in the dark for 7–42 days. After cultivation, the fungal colonized wood pieces, and the mycelial mats from the liquid malt extract cultivations were separately harvested and immediately frozen with liquid nitrogen, and stored at −80 °C prior to RNA and protein extractions.

### RNA extraction and purification

The frozen samples from two biological replicate wood cultivations (2 and 4 weeks of growth) and mycelial mats (2 weeks of growth on malt extract medium) were used for RNA extraction. The 2-g wood samples were ground under liquid nitrogen with A11 Basic analytical mill (IKA), and total RNA was extracted by CsCl gradient centrifugation method [81]. The quantity and quality of the dialyzed RNA fractions were estimated by using Agilent 2100 Bioanalyzer (Agilent Technologies) with the RNA6000 Nano Assay. Poly-A mRNA was further purified from the RNA fractions of accepted quality using Dynabeads mRNA Purification Kit (Invitrogen) by following manufacturer’s instructions. The purified mRNA fractions were quantified by using NanoDrop1000 Spectrophotometer (Thermo Scientific) and Qubit Fluorometer (Thermo Fisher Scientific).

### Illumina RNA sequencing and data treatments

From each mRNA fraction, a library was constructed using a TruSeq Stranded mRNA Library Prep Kit according to the manufacturer’s instructions (Illumina, Inc.). The libraries were paired-end sequenced using MiSeq (326 + 286 bp) and NextSeq 500 (86 + 74 bp) sequencers (Illumina, Inc.). Pre-processing of the reads was performed with Cutadapt version 1.7.1. Adapters were removed and reads were quality trimmed from the 3′ ends. Only reads that fulfilled the pairing criteria (both R1 and R2 reads present) and were >50 bp in length were included in the analysis.

In average, approximately 93 % of the raw reads were left in each sample after data filtering and cleaning. RNA-seq reads were mapped against gene models of the genomic assembly of *P. radiata* (to be published elsewhere) by STAR aligner version 2.4.1b [82]. Alignments were guided by an annotation file containing the genomic coordinates of gene models predicted by the BRAKER1 software [83]. Resulting alignment files were cleaned using Bamtools.

Aligned reads were counted using HTSeq software [84] guided by the annotation file. Read counts were transformed by variance stabilizing transformation (VST) method after which principal component analysis (PCA) and hierarchical clustering of the samples were performed using DESeq2 package [85]. Differential expression was as well analyzed in DESeq2 package [85]. Significantly differentially expressed genes were identified using thresholds of Benjamini–Hochberg adjusted *p* < 0.05 and log2-fold change ≥1 or ≤−1. HTSeq and DESeq2 analyses as well as construction of the PCA and heatmap were performed in the Chipster platform [86].

Transcripts were functionally annotated by PANNZER software [87] and Blastp (version 2.2.30) searches against the NCBI non-redundant protein sequences database [88]. For visualization and clustering purposes, read counts were transformed by regularized log transformation (rlog) method of the DESeq2 package [85, 86]. Hierarchical clustering and visualization was performed using heatmap.2 function within gplots package of the R environment [89, 90].

### Protein extraction and purification

Three parallel fungal cultivations on spruce wood after 7, 14, 21, 28, and 42 days were used for studying the total proteome of *P. radiata*. Spruce wood after adding the inoculum (0 day cultivation) and without the inoculum were used as controls. Spruce sticks from one culture flask (2 g) were ground with A11 Basic analytical mill (IKA) under liquid nitrogen. The milled wood was transferred to 40 ml of 25 mM potassium phosphate buffer (pH 7) containing 0.01 % (v/v) Tween80 and 0.2 mM phenylmethane sulfonyl fluoride (PMSF) as protease inhibitor. This mixture was incubated on a magnetic stirrer for 4 h at 4 °C, and the liquid was separated by filtering through glass fiber filters (GF/C, Whatman) under suction.

After filtration the samples were stored at −20 °C overnight. Thawed samples were precipitated by direct addition of solid trichloroacetic acid (TCA) to 10 % (w/v). Following overnight storage at −20 °C, the precipitate was centrifuged at 5000 g for 15 min at 4 °C, and the pellet was washed three times with cold acetone. To solubilize the air-dried protein pellet, 8.0 M urea was added, followed by overnight mixing and sonication (two times for 1 h). This suspension was centrifuged two times at 21,000 *g* for 15 min, and the supernatant was collected and diluted to a final concentration of 1.5 M urea. The cystein–cystein covalent bonds of the proteins in the samples were reduced with 0.05 M dithiothreitol (Sigma-Aldrich, USA) for 20 min at 37 °C, and then alkylated with 0.15 M iodoacetamide (Fluka, Sigma-Aldrich, USA) at room temperature. Samples were digested by adding 0.75 µg sequencing grade trypsin (Promega, USA), and incubated for overnight at 37 °C. Resulting peptides were purified two times with C18 Microspin columns (Harvard Apparatus) according to the protocol of the manufacturer, and redissolved in 50 µl of A-buffer (0.1 % m/vol TFA (trifluoroacetic acid) in 1 % vol/vol acetonitrile solution in HPLC grade water).

### Protein identification by peptide LC–MS/MS

Liquid chromatography coupled to tandem mass spectrometry (LC–MS/MS) analysis was carried out on an EASY-n1000 HPLC (Thermo Fisher Scientific, Germany) connected to a Q Exactive hybrid quadrupole orbitrap mass spectrometer (Thermo Fisher Scientific, Germany) with nano-electrospray ion source (Thermo Fisher Scientific, Germany). The LC–MS/MS samples were separated using a two-column set-up consisting of an Acclaim PepMap 100 pre-column C18, 3 μm, 100 Å; ID 75 μm × 2 cm) and separated with an Acclaim PepMap RSLC analytical column (C18, 2 μm, 100 Å; ID 55 μm × 15 cm (Thermo Fisher Scientific, Germany). The linear separation gradient consisted of 5 % buffer B in 5 min, 35 % buffer B in 60 min, 80 % buffer B in 5 min and 100 % buffer B in 10 min at a flow rate of 0.3 µl/min (buffer A: 0.1 % FA (fluoroacetic acid), 0.01 % TFA in 1 % acetonitrile; buffer B: 0.1 % FA, 0.01 % TFA in 98 % acetonitrile). Peptide samples (100-fold diluted, 2 µl volume) were injected for each LC–MS/MS run and analyzed. Full MS scan was acquired with a resolution of 60,000 at normal mass range in the orbitrap analyzer. The method was set to fragment the 10 most intense precursor ions with higher energy collisional dissociation (energy 28). Data were acquired using Q Exactive Tune software (Thermo Fisher Scientific, Germany).

Proteins were identified and quantified using Andromeda search engine combined with MaxQuant proteomics software [91, 92]. Raw data were searched against the translated coding sequence gene models of *P. radiata* complemented with trypsin and tag sequences. Database searches were limited to fully tryptic peptides with a maximum of two missed cleavages. Cysteine carbamidomethylation and methionine oxidation were set as fixed and variable modifications, respectively. Error tolerances on the precursor and fragment ions were ± 4.5 ppm and ± 0.5 Da, respectively. Results were filtered to a maximum false discovery rate (FDR) of 0.05. For each spectrum, a propensity score matching (PSM) with high score was retained and these PSMs were further filtered with the cutoff of Andromeda score (>40) and delta score (>8). The peptide FDR was set to <0.01.

### Protein quantitation and functional prediction of the proteome

For abundance calculation, mass spectrometric signal intensities (MaxQuant) of peptide precursor ions belonging to each protein were divided by the total abundance of all detected proteins at each time point and for each wood culture. Protein abundance values and their standard deviation were calculated from the normalized values of the three biological replicate cultures. In addition, consistency of the biological replicates was studied by PCA. The abundance values were subjected to arcsin transformation, and PCA was performed with stats package of the R environment and visualized with ggbiplot package [89, 93]. Cellular locations of LC–MS/MS identified proteins being transmembrane, intracellular or extracellular, were analyzed with Phobius predictor [32] together with in silico-predicted secretome, the latter being based on identification of potential secretion signals in the translated protein models corresponding to respective gene models in *P. radiata* genome. Gene ontology (GO) annotations for LC–MS/MS identified proteins were assigned using Blast2GO software [94] and annotations of CAZy-encoding genes were subjected to manual quality control. Peptidases were classified according to Blastp searches against the MEROPS database (http://merops.sanger.ac.uk/ [95]).

### Enzyme activity and protein concentration measurements

Enzyme activities and protein concentrations were measured from the solution phase (filtered phosphate buffer with solutes) from above-mentioned proteome samples in which the ground wood samples were incubated before TCA precipitation. Measurements of laccase, manganese peroxidase, xylanase, β-glucosidase, and cellobiohydrolase activities were performed as previously described [18]. Protein concentrations were measured by the BCA Protein Assay (Pierce, Thermo Fisher Scientific, Rockford, USA) according manufacturer’s instructions and using bovine serum albumin as reference protein.

### Lignin composition analysis of fungal-colonized spruce wood

Similar to the transcriptome and proteome experiments, the solid spruce wood cultures were harvested after 42 days of cultivation, together with reference wood samples (non-inoculated wood sticks without fungus were similarly incubated for 42 days) and dried at 90 °C for 72 h. After this, the dried wood pieces were ground with A11 Basic analytical mill (IKA, Germany) and sieved through a 1-mm particle size metal screen for homogeneity.

Gravimetric (Klason) lignin and acid soluble lignin were analyzed from three biological cultivation and two technical replicate 0.1-g samples. The ground wood samples were mixed with 2 ml of 72 % sulfuric acid (Sigma-Aldrich, Germany) using a magnetic stirrer for 2 h at room temperature. After mixing, the total volume was adjusted to 50 ml with deionized water, and the mixtures were autoclaved at 121 °C, 1 atm, for 30 min. The cooled samples were filtered through 30-ml glass filter crucible (porosity 4, ROBU Glasfilter-Geraete GmbH, Germany) with vacuum suction. The insoluble residue was washed with 35 ml of deionized water and dried in an oven overnight at 90 °C. Filtrates were adjusted to 100 ml with deionized water. The acid soluble lignin was determined spectrophotometrically at 205 nm (absorption coefficient 128 g cm^−1^ l^−1^, [96]) with Shimadzu Pharma-Spec UV-1700 spectrophotometer. The dry mass of solids was weighted for obtaining the Klason lignin content of the samples.

The same fractions (0.2 g of each) were further ground in a planetary ball mill (Fritsch Planetary Mono Mill Pulverisette, Fritsch GmbH, Germany) using tungsten-carbide cup (29 ml) with four balls. The milling time was 25 min with milling frequency 350 rpm, after which a 20 min pause was introduced to prevent overheating. The cycle was repeated seven times with overall milling time of 5 h. The analytical scale Py–GC/MS equipment Pyrolab2000 (Pyrolab, Sweden) was adopted, and three to four different runs were performed on each sample using a platinum foil pulse pyrolyzer and 580 °C isothermal pyrolysis temperature [97]. The system was directly connected to Bruker Scion SQ 456-GC/MS equipped with Agilent DB-5MS UI (5 %-phenyl)-methylpolysiloxane, 30 m × 0.250 mm × 0.25 µm film) capillary column. The injector temperature was 250 °C, ion source 250 °C with electron ionization of 70 eV, the MS scan range m/z 40–400 and helium as carrier gas at the flow rate of 1 mL/min and 1:20 split ratio. From the base line corrected GC/MS total ion count (TIC) chromatograms, the amount of aromatic degradation products as area was compared to the total area (relative peak areas). The products used in calculations were measured between retention time of 3.04–20 min, and 78 peaks were included in quantification. The samples were treated equally, and the pyrolysis was performed under the same conditions. Products were identified by selected reference compounds with their retention times and mass spectra, and by comparison with National Institute of Standards and Technology (NIST) library and with literature [97–100].

### Electron microscopy of wood decay

Spruce wood sticks from 42 days cultivated solid-state cultures of *P. radiata* were inspected with field emission scanning electron microscopy (FE-SEM) to visually study the fungal hyphal growth in wood, and wood decay processing. Vertical and transverse sections of wood sticks were cut with a sharp blade and fixed according to Ji et al. [43] except that the dehydration was performed with an increasing series of ethanol (from 20 to 98 %, v/v) and acetone (30 to 90 %, v/v). After freeze-drying, the samples were coated with a few nm layer of Au/Pd alloy using a Cressington 208 HR high-resolution sputter coater (Cressington, UK) before imaging with Hitachi S-4800 FE-SEM (Hitachi, Japan).

## Additional files

10.1186/s13068-016-0608-9 Complete list of gene models, their locations in the genome assembly, transcript counts and normalized abundances, peptide and protein abundances, and functional annotations, of the transcriptome and proteome of *P. radiata* cultivated on spruce wood.
10.1186/s13068-016-0608-9 Principal component analysis A) for the normalized protein abundance values of three biological replicates extracted at the weekly time points (0–6 weeks) from wood cultivations, B) for normalized transcript count values of two RNA-sequencing biological replicates from 2 week time point (2 weeks) of malt extract cultivations and 2 and 4 week (4 weeks) time points of wood cultivations, C) hierarchical clustering of the transcriptome samples according to normalized count values. Ellipses in A) represent general trend of the groups with 68 % confidence interval.
10.1186/s13068-016-0608-9 Protein production and CAZyme activities from protein extracts of the solid-state cultures of *P. radiata* during 6 weeks of growth on spruce wood. Mean values with standard deviations of three biological replicate cultures are presented.
10.1186/s13068-016-0608-9 Number of *P. radiata* CAZy and AA enzyme encoding transcripts, and detected as proteins in the proteome analyses, and upregulated on spruce wood cultivations. Values include all proteins identified in the wood cultivation proteomes by peptide LC–MS/MS analysis. Only proteins with equal or more than two unique peptides were included. Upregulated transcripts: *p* < 0.05 and log2-fold change ≥1 at both (2-week and 4-week) time points.
10.1186/s13068-016-0608-9 Summary of lignocellulose acting proteins identified in the spruce wood cultivation proteomes of *P. radiata* by peptide LC–MS/MS analysis.

## Abbreviations

AA: auxiliary activity enzyme
ALE: aldose-1-epimerase
AOX: alcohol oxidase
CAZy, CAZyme: carbohydrate-active enzyme
CBM: carbohydrate binding module
CDH: cellobiose dehydrogenase
CE: carbohydrate esterase
CRO: copper radical oxidase
DyP: dye-decolorizing peroxidase
FE-SEM: field emission scanning electron microscopy
GC/MS: gas chromatography/mass spectrometry detection
GH: glycoside hydrolase
GLOX: glyoxal oxidase
GMC: glucose–methanol–choline
GO: gene ontology
GT: glycosyltransferase
Lacc: laccase
LPMO: lytic polysaccharide monooxygenase
LiP: lignin peroxidase
MnP: manganese peroxidase
ORF: open reading frame
Ph: phenyl
PL: polysaccharide lyase
TCA: trichloroacetic acid

## References

[1] Floudas D, Binder M, Riley R, Barry K, Blanchette RA, Henrissat B (2012). The Paleozoic origin of enzymatic lignin decomposition reconstructed from 31 fungal genomes. Science.

[2] Lundell TK, Mäkelä MR, de Vries RP, Hildén KS, Francis MM (2014). Genomics, lifestyles and future prospects of wood-decay and litter-decomposing Basidiomycota. Advances in botanical research.

[3] Riley R, Salamov AA, Brown DW, Nagy LG, Floudas D, Held BW (2014). Extensive sampling of basidiomycete genomes demonstrates inadequacy of the white-rot/brown-rot paradigm for wood decay fungi. Proc Natl Acad Sci USA.

[4] Eastwood DC, Floudas D, Binder M, Majcherczyk A, Schneider P, Aerts A (2011). The plant cell wall-decomposing machinery underlies the functional diversity of forest fungi. Science.

[5] Hatakka A, Hammel KE, Hofrichter M (2010). Fungal biodegradation of lignocelluloses. Industrial applications, the mycota X.

[6] Martínez ÁT, Ruiz-Dueñas FJ, Martínez MJ, del Río JC, Gutiérrez A (2009). Enzymatic delignification of plant cell wall: from nature to mill. Curr Opin Biotechnol.

[7] Vanden Wymelenberg A, Gaskell J, Mozuch M, Kersten P, Sabat G, Martinez D (2009). Transcriptome and secretome analyses of *Phanerochaete chrysosporium* reveal complex patterns of gene expression. Appl Environ Microbiol.

[8] Levasseur A, Drula E, Lombard V, Coutinho PM, Henrissat B (2013). Expansion of the enzymatic repertoire of the CAZy database to integrate auxiliary redox enzymes. Biotechnol Biofuels.

[9] Ruiz-Dueñas FJ, Lundell T, Floudas D, Nagy LG, Barrasa JM, Hibbett DS (2013). Lignin-degrading peroxidases in Polyporales: an evolutionary survey based on 10 sequenced genomes. Mycologia.

[10] Hofrichter M, Ullrich R, Pecyna MJ, Liers C, Lundell T (2010). New and classic families of secreted fungal heme peroxidases. Appl Microbiol Biotechnol.

[11] Kersten P, Cullen D (2014). Copper radical oxidases and related extracellular oxidoreductases of wood-decay agaricomycetes. Fungal Genet Biol.

[12] Ferreira P, Carro J, Serrano A, Martínez AT (2015). A survey of genes encoding H_2_O_2_-producing GMC oxidoreductases in 10 Polyporales genomes. Mycologia.

[13] Sugano Y (2009). DyP-type peroxidases comprise a novel heme peroxidase family. Cell Mol Life Sci.

[14] Rytioja J, Hildén K, Yuzon J, Hatakka A, de Vries RP, Mäkelä MR (2014). Plant-polysaccharide-degrading enzymes from Basidiomycetes. Microbiol Mol Biol Rev.

[15] Fernández-Fueyo E, Ruiz-Dueñas FJ, Miki Y, Martínez MJ, Hammel KE, Martínez AT (2012). Lignin-degrading peroxidases from genome of selective ligninolytic fungus *Ceriporiopsis subvermispora*. J Biol Chem.

[16] Vanden Wymelenberg A, Gaskell J, Mozuch M, BonDurant SS, Sabat G, Ralph J (2011). Significant alteration of gene expression in wood decay fungi *Postia placenta* and *Phanerochaete chrysosporium* by plant species. Appl Environ Microbiol.

[17] Skyba O, Cullen D, Douglas CJ, Mansfield D (2016). Gene expression patterns of wood decay fungi *Postia placenta* and *Phanerochaete chrysosporium* are influenced by wood substrate composition during degradation. Appl Environ Microbiol.

[18] Kuuskeri J, Mäkelä MR, Isotalo J, Oksanen I, Lundell T (2015). Lignocellulose-converting enzyme activity profiles correlate with molecular systematics and phylogeny grouping in the incoherent genus *Phlebia* (Polyporales, Basidiomycota). BMC Microbiol.

[19] Binder M, Justo A, Riley R, Salamov A, Lopez-Giraldez F, Sjökvist E (2013). Phylogenetic and phylogenomic overview of the Polyporales. Mycologia.

[20] Nakasone KK, Sytsma KJ (1993). Biosystematic studies on *Phlebia acerina*, *P. rufa*, and *P. radiata* in North America. Mycologia.

[21] Ghobad-Nejhad M, Hallenberg N (2010). Multiple evidence for recognition of *Phlebia tuberculata*, a more widespread segregate of *Phlebia livida* (Polyporales, Basidiomycota). Mycol Prog.

[22] Hakala TK, Maijala P, Konn J, Hatakka A (2004). Evaluation of novel wood-rotting polypores and corticioid fungi for the decay and biopulping of Norway spruce (*Picea abies*) wood. Enzyme Microb Technol.

[23] Peltola A (2014). Finnish statistical yearbook of forestry. Finnish forest research institute.

[24] Hildén KS, Mäkelä MR, Hakala TK, Hatakka A, Lundell T (2006). Expression on wood, molecular cloning and characterization of three lignin peroxidase (LiP) encoding genes of the white rot fungus *Phlebia radiata*. Curr Genet.

[25] Hildén K, Martinez AT, Hatakka A, Lundell T (2005). The two manganese peroxidases Pr-MnP2 and Pr-MnP3 of *Phlebia radiata*, a lignin-degrading basidiomycete, are phylogenetically and structurally divergent. Fungal Genet Biol.

[26] Saloheimo M, Niku-Paavola ML, Knowles JK (1991). Isolation and structural analysis of the laccase gene from the lignin-degrading fungus *Phlebia radiata*. J Gen Microbiol.

[27] Mäkelä MR, Hildén KS, Hakala TK, Hatakka A, Lundell TK (2006). Expression and molecular properties of a new laccase of the white rot fungus *Phlebia radiata* grown on wood. Curr Genet.

[28] Hofrichter M, Lundell T, Hatakka A (2001). Conversion of milled pine wood by manganese peroxidase from *Phlebia radiata*. Appl Environ Microbiol.

[29] Lundell T, Bentley E, Hildén K, Rytioja J, Kuuskeri J, Ufot UF (2016). Engineering towards catalytic use of fungal class-II peroxidases for dye-decolorizing and conversion of lignin model compounds. Curr Biotechnol.

[30] Lundell T, Wever R, Floris R, Harvey P, Hatakka A, Brunow G (1993). Lignin peroxidase L3 from *Phlebia radiata*. Pre-steady-state and steady-state studies with veratryl alcohol and a non-phenolic lignin model compound 1-(3,4-dimethoxyphenyl)-2-(2-methoxyphenoxy)propane-1,3-diol. Eur J Biochem.

[31] Hildén KS, Bortfeldt R, Hofrichter M, Hatakka A, Lundell TK (2008). Molecular characterization of the basidiomycete isolate *Nematoloma frowardii* b19 and its manganese peroxidase places the fungus in the corticioid genus *Phlebia*. Microbiology.

[32] Käll L, Krogh A, Sonnhammer ELL (2004). A combined transmembrane topology and signal peptide prediction method. J Mol Biol.

[33] Manavalan A, Adav SS, Sze SK (2011). ITRAQ-based quantitative secretome analysis of *Phanerochaete chrysosporium*. J Proteomics.

[34] Gaskell J, Marty A, Mozuch M, Kersten PJ, Splinter BonDurant S, Sabat G (2014). Influence of *Populus* genotype on gene expression by the wood decay fungus *Phanerochaete chrysosporium*. Appl Environ Microbiol.

[35] Mahajan S, Master ER (2010). Proteomic characterization of lignocellulose-degrading enzymes secreted by *Phanerochaete carnosa* grown on spruce and microcrystalline cellulose. Appl Microbiol Biotechnol.

[36] Hori C, Ishida T, Igarashi K, Samejima M, Suzuki H, Master E (2014). Analysis of the *Phlebiopsis gigantea* genome, transcriptome and secretome provides insight into its pioneer colonization strategies of wood. PLoS Genet.

[37] Salvachúa D, Martínez AT, Tien M, López-Lucendo MF, García F, de Los Ríos V (2013). Differential proteomic analysis of the secretome of *Irpex lacteus* and other white-rot fungi during wheat straw pretreatment. Biotechnol Biofuels.

[38] Manavalan T, Manavalan A, Thangavelu KP, Heese K (2012). Secretome analysis of *Ganoderma lucidum* cultivated in sugarcane bagasse. J Proteomics.

[39] Hori C, Gaskell J, Igarashi K, Kersten P, Mozuch M, Samejima M (2014). Temporal alterations in the secretome of the selective ligninolytic fungus *Ceriporiopsis subvermispora* during growth on aspen wood reveal this organism’s strategy for degrading lignocellulose. Appl Environ Microbiol.

[40] Zhu N, Liu J, Yang J, Lin Y, Yang Y, Ji L (2016). Comparative analysis of the secretomes of *Schizophyllum commune* and other wood-decay basidiomycetes during solid–state fermentation reveals its unique lignocellulose–degrading enzyme system. Biotechnol Biofuels.

[41] Levasseur A, Lomascolo A, Chabrol O, Ruiz-Dueñas FJ, Boukhris-Uzan E, Piumi F (2014). The genome of the white-rot fungus *Pycnoporus cinnabarinus*: a basidiomycete model with a versatile arsenal for lignocellulosic biomass breakdown. BMC Genomics.

[42] Couturier M, Navarro D, Chevret D, Henrissat B, Piumi F, Ruiz-Dueñas FJ (2015). Enhanced degradation of softwood versus hardwood by the white-rot fungus *Pycnoporus coccineus*. Biotechnol Biofuels.

[43] Ji XL, Zhang WT, Gai YP, Lu BY, Yuan CZ, Liu QX (2012). Patterns of lignocellulose degradation and secretome analysis of *Trametes trogii* MT. Int Biodeterior Biodegrad.

[44] Alfaro M, Castanera R, Lavín JL, Grigoriev IV, Oguiza JA, Ramírez L (2016). Comparative and transcriptional analysis of the predicted secretome in the lignocellulose-degrading basidiomycete fungus *Pleurotus ostreatus*. Environ Microbiol.

[45] Fernández-Fueyo E, Ruiz-Dueñas FJ, López-Lucendo MF, Pérez-Boada M, Rencoret J, Gutiérrez A (2016). A secretomic view of woody and nonwoody lignocellulose degradation by *Pleurotus ostreatus*. Biotechnol Biofuels.

[46] Floudas D, Held BW, Riley R, Nagy LG, Koehler G, Ransdell AS (2015). Evolution of novel wood decay mechanisms in Agaricales revealed by the genome sequences of *Fistulina hepatica* and *Cylindrobasidium torrendii*. Fungal Genet Biol.

[47] Korripally P, Hunt CG, Houtman CJ, Jones DC, Kitin PJ, Cullen D (2015). Regulation of gene expression during the onset of ligninolytic oxidation by *Phanerochaete chrysosporium* on spruce wood. Appl Environ Microbiol.

[48] Niku-Paavola M-L, Karhunen E, Kantelinen A, Viikari L, Lundell T, Hatakka A (1990). The effect of culture conditions on the production of lignin modifying enzymes by the white-rot fungus *Phlebia radiata*. J Biotechnol.

[49] Vares T, Kalsi M, Hatakka A (1995). Lignin peroxidases, manganese peroxidases, and other ligninolytic enzymes produced by *Phlebia radiata* during solid-state fermentation of wheat straw. Appl Environ Microbiol.

[50] Kersten PJ (1990). Glyoxal oxidase of *Phanerochaete chrysosporium*: its characterization and activation by lignin peroxidase. Proc Natl Acad Sci USA.

[51] Hammel K, Mozuch MD, Jensen KA, Kersten PJ (1994). Recycling during oxidation of the arylglycerol p-aryl ether lignin structure. Biochemistry.

[52] Hernández-Ortega A, Ferreira P, Martínez AT (2012). Fungal aryl-alcohol oxidase: a peroxide-producing flavoenzyme involved in lignin degradation. Appl Microbiol Biotechnol.

[53] Mäkelä MR, Lundell T, Hatakka A, Hildén K (2013). Effect of copper, nutrient nitrogen, and wood-supplement on the production of lignin-modifying enzymes by the white-rot fungus *Phlebia radiata*. Fungal Biol.

[54] Niku-Paavola ML, Karhunen E, Salola P, Raunio V (1988). Ligninolytic enzymes of the white-rot fungus *Phlebia radiata*. Biochem J.

[55] Lundell T, Hatakka A (1994). Participation of Mn(II) in the catalysis of laccase, manganese peroxidase and lignin peroxidase from *Phlebia radiata*. FEBS Lett.

[56] Lundell T, Leonowicz A, Rogalski J, Hatakka A (1990). Formation and action of lignin-modifying enzymes in cultures of *Phlebia radiata* supplemented with veratric acid. Appl Environ Microbiol.

[57] Martinez D, Larrondo LF, Putnam N, Gelpke MDS, Huang K, Chapman J (2004). Genome sequence of the lignocellulose degrading fungus *Phanerochaete chrysosporium* strain RP78. Nat Biotechnol.

[58] Suzuki H, MacDonald J, Syed K, Salamov A, Hori C, Aerts A (2012). Comparative genomics of the white-rot fungi, *Phanerochaete carnosa* and *P. chrysosporium*, to elucidate the genetic basis of the distinct wood types they colonize. BMC Genomics.

[59] Xie C, Luo W, Li Z, Yan L, Zhu Z, Wang J (2016). Secretome analysis of *Pleurotus eryngii* reveals enzymatic composition for ramie stalk degradation. Electrophoresis.

[60] Langston JA, Shaghasi T, Abbate E, Xu F, Vlasenko E, Sweeney MD (2011). Oxidoreductive cellulose depolymerization by the enzymes cellobiose dehydrogenase and glycoside hydrolase 61. Appl Environ Microbiol.

[61] Vaaje-Kolstad G, Westereng B, Horn SJ, Liu Z, Zhai H, Sørlie M (2010). An Oxidative enzyme boosting the enzymatic conversion of recalcitrant polysaccharides. Science.

[62] Agger JW, Isaksen T, Várnai A, Vidal-Melgosa S, Willats WGT, Ludwig R (2014). Discovery of LPMO activity on hemicelluloses shows the importance of oxidative processes in plant cell wall degradation. Proc Natl Acad Sci USA.

[63] Kracher D, Scheiblbrandner S, Felice AKG, Breslmayr E, Preims M, Haltrich D (2016). Extracellular electron transfer systems fuel cellulose oxidative degradation. Science.

[64] Courtade G, Wimmer R, Røhr ÅK, Preims M, Felice AKG, Dimarogona M (2016). Interactions of a fungal lytic polysaccharide monooxygenase with β-glucan substrates and cellobiose dehydrogenase. Proc Natl Acad Sci USA.

[65] Yoshida M, Igarashi K, Wada M, Kaneko S, Suzuki N, Matsumura H (2005). Characterization of carbohydrate-binding cytochrome b 562 from the white-rot fungus *Phanerochaete chrysosporium*. Appl Environ Microbiol.

[66] Henriksson G, Johansson G, Pettersson G (2000). A critical review of cellobiose dehydrogenases. J Biotechnol.

[67] Hori C, Gaskell J, Igarashi K, Samejima M, Hibbett D, Henrissat B (2013). Genomewide analysis of polysaccharides degrading enzymes in 11 white- and brown-rot Polyporales provides insight into mechanisms of wood decay. Mycologia.

[68] Vanden Wymelenberg A, Sabat G, Martinez D, Rajangam AS, Teeri TT, Gaskell J (2005). The *Phanerochaete chrysosporium* secretome: database predictions and initial mass spectrometry peptide identifications in cellulose-grown medium. J Biotechnol.

[69] Sato S, Liu F, Koc H, Tien M (2007). Expression analysis of extracellular proteins from *Phanerochaete chrysosporium* grown on different liquid and solid substrates. Microbiology.

[70] Keller B, Templeton MD, Lamb CJ (1989). Specific localization of a plant cell wall glycine-rich protein in protoxylem cells of the vascular system. Proc Natl Acad Sci USA.

[71] Eriksson KE, Pettersson B (1982). Purification and partial characterization of two acidic proteases from the white-rot fungus *Sporotrichum pulverulentum*. Eur J Biochem.

[72] Habu N, Samejima M, Dean JFD, Eriksson KEL (1993). Release of the FAD domain from cellobiose oxidase by proteases from cellulolytic cultures of *Phanerochaete chrysosporium*. FEBS Lett.

[73] Dosoretz CG, Chen H, Grethlein HE (1990). Effect of environmental conditions on extracellular protease activity in ligninolytic cultures of *Phanerochaete chrysosporium*. Appl Environ Microbiol.

[74] Dosoretz CG, Dass SB, Reddy CA, Grethlein HE (1990). Protease-mediated degradation of lignin peroxidase in liquid cultures of *Phanerochaete chrysosporium*. Appl Environ Microbiol.

[75] Alfaro M, Oguiza JA, Ramírez L, Pisabarro AG (2014). Comparative analysis of secretomes in basidiomycete fungi. J Proteomics.

[76] Adav SS, Ravindran A, Chao LT, Tan L, Singh S, Sze SK (2011). Proteomic analysis of pH and strains dependent protein secretion of *Trichoderma reesei*. J Proteome Res.

[77] Häkkinen M, Sivasiddarthan D, Aro N, Saloheimo M, Pakula TM (2015). The effects of extracellular pH and of the transcriptional regulator PACI on the transcriptome of *Trichoderma reesei*. Microb Cell Fact.

[78] Eriksson KE, Blanchette RA, Ander P (1990). Microbial and enzymatic degradation of wood and wood components.

[79] Ravalason H, Jan G, Mollé D, Pasco M, Coutinho PM, Lapierre C (2008). Secretome analysis of *Phanerochaete chrysosporium* strain CIRM-BRFM41 grown on softwood. Appl Microbiol Biotechnol.

[80] Singh D, Zeng J, Laskar DD, Deobald L, Hiscox WC, Chen S (2011). Investigation of wheat straw biodegradation by *Phanerochaete chrysosporium*. Biomass Bioenergy.

[81] Patyshakuliyeva A, Mäkelä MR, Sietiö O-M, de Vries RP, Hildén KS (2014). An improved and reproducible protocol for the extraction of high quality fungal RNA from plant biomass substrates. Fungal Genet Biol.

[82] Dobin A, Davis CA, Schlesinger F, Drenkow J, Zaleski C, Jha S (2013). STAR: ultrafast universal RNA-seq aligner. Bioinformatics.

[83] Hoff KJ, Lange S, Lomsadze A, Borodovsky M, Stanke M (2015). BRAKER1: unsupervised RNA-Seq-based genome annotation with GeneMark-ET and AUGUSTUS. Bioinformatics.

[84] Anders S, Pyl PT, Huber W (2015). HTSeq-A Python framework to work with high-throughput sequencing data. Bioinformatics.

[85] Love MI, Huber W, Anders S (2014). Moderated estimation of fold change and dispersion for RNA-seq data with DESeq2. Genome Biol.

[86] Kallio MA, Tuimala JT, Hupponen T, Klemelä P, Gentile M, Scheinin I (2011). Chipster: user-friendly analysis software for microarray and other high-throughput data. BMC Genomics.

[87] Koskinen P, Törönen P, Nokso-Koivisto J, Holm L (2014). PANNZER: high-throughput functional annotation of uncharacterized proteins in an error-prone environment. Bioinformatics.

[88] Altschul SF, Madden TL, Schaffer AA, Zhang J, Zhang Z, Miller W (1997). Gapped BLAST and PSI-BLAST: a new generation of protein database search programs. Nucleic Acids Res.

[89] R Core Team. R: a language and environment for statistical computing. Vienna: R foundation for statistical computing; 2015. http://www.r-project.org/. Accessed 18 Aug 2016.

[90] Warnes GR, Bolker B, Bonebakker L, Gentleman R, Liaw WHA, Lumley T, et al. gplots: various R programming tools for plotting data. 2015. https://www.cran.r-project.org/package=gplots. Accessed 18 Aug 2016.

[91] Cox J, Mann M (2008). MaxQuant enables high peptide identification rates, individualized p.p.b.-range mass accuracies and proteome-wide protein quantification. Nat Biotechnol.

[92] Cox J, Neuhauser N, Michalski A, Scheltema RA, Olsen JV, Mann M (2011). Andromeda: a peptide search engine integrated into the MaxQuant environment. J Proteome Res.

[93] Vu VQ. Ggbiplot: A ggplot2 based biplot. R package version 0.55. 2011. http://www.github.com/vqv/ggbiplot. Accessed 18 Aug 2016.

[94] Conesa A, Götz S, García-Gómez JM, Terol J, Talón M, Robles M (2005). Blast2GO: a universal tool for annotation, visualization and analysis in functional genomics research. Bioinformatics.

[95] Rawlings ND, Barrett AJ, Finn R (2016). Twenty years of the MEROPS database of proteolytic enzymes, their substrates and inhibitors. Nucleic Acids Res.

[96] Iakovlev M, van Heiningen A (2011). SO_2_-ethanol-water (SEW) pulping: I. Lignin determination in pulps and liquors. J Wood Chem Technol.

[97] Ohra-Aho T, Tenkanen M, Tamminen T (2005). Direct analysis of lignin and lignin-like components from softwood kraft pulp by Py-GC/MS techniques. J Anal Appl Pyrolysis.

[98] Alves A, Schwanninger M, Pereira H, Rodrigues J (2006). Analytical pyrolysis as a direct method to determine the lignin content in wood: Part 1: Comparison of pyrolysis lignin with Klason lignin. J Anal Appl Pyrolysis.

[99] Gu X, Ma X, Li L, Liu C, Cheng K, Li Z (2013). Pyrolysis of poplar wood sawdust by TG-FTIR and Py-GC/MS. J Anal Appl Pyrolysis.

[100] Lupoi JS, Singh S, Parthasarathi R, Simmons BA, Henry RJ (2015). Recent innovations in analytical methods for the qualitative and quantitative assessment of lignin. Renew Sustain Energ Rev.

