# Supplementary figures and images for: Time-scale dynamics of proteome and transcriptome of the white-rot fungus Phlebia radiata: growth on spruce wood and decay effect on lignocellulose
Source: Biotechnol Biofuels. 2016 Sep 5;9(1):192. doi: 10.1186/s13068-016-0608-9 (PMC5011852; doi:10.1186/s13068-016-0608-9)

A

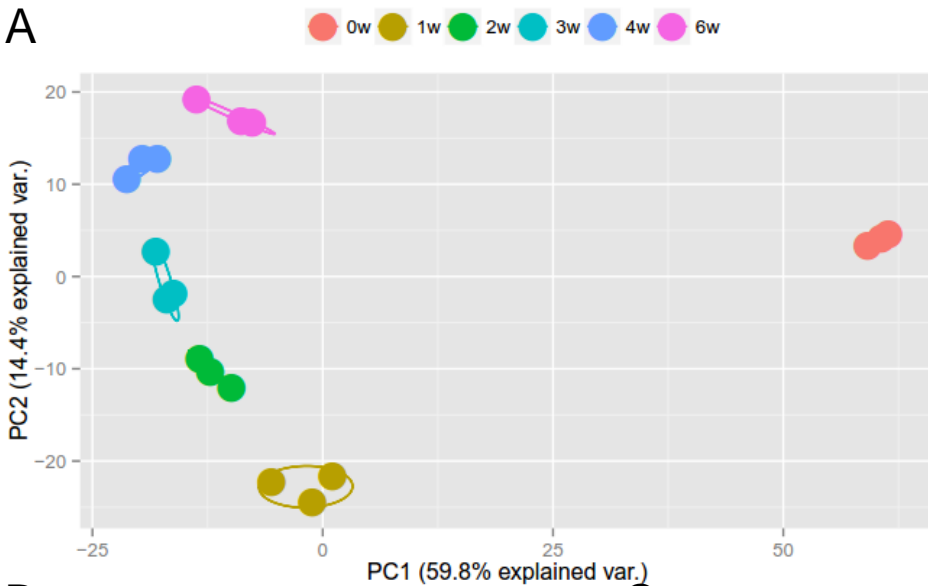

B

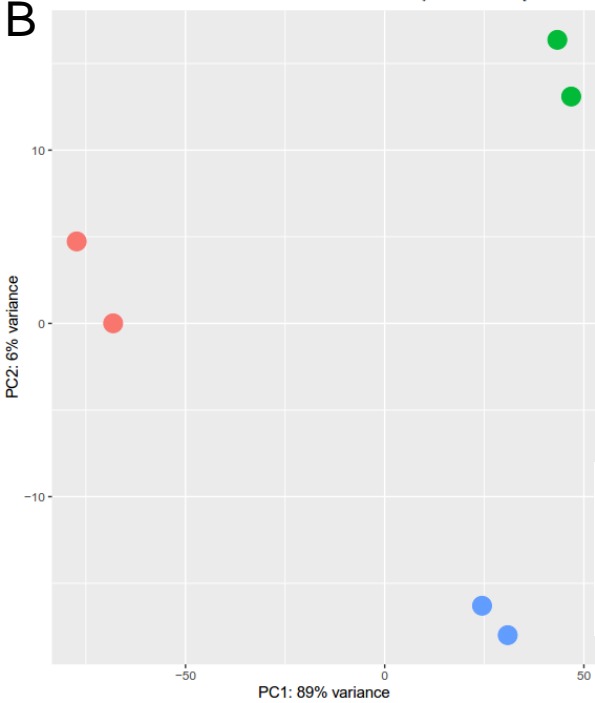

C

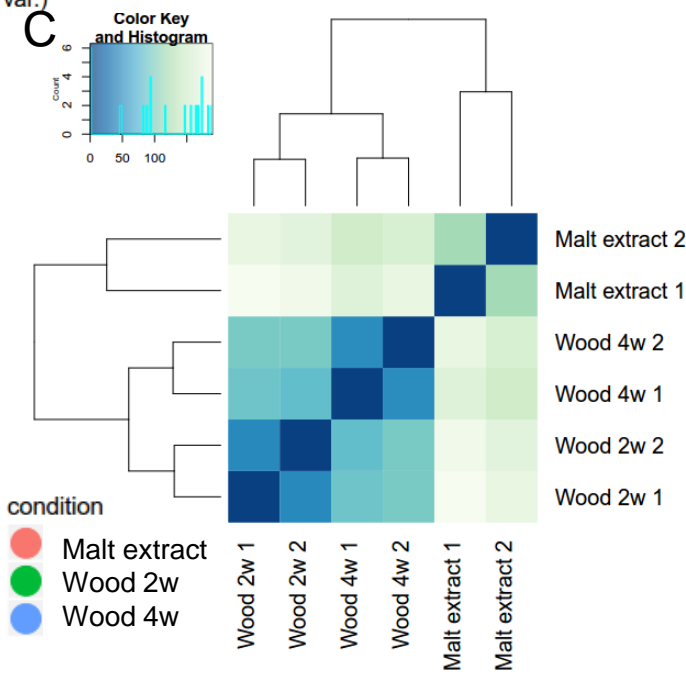

Supplement: Supplementary file 2 — 10.1186/s13068-016-0608-9 Principal component analysis A) for the normalized protein abundance values of three biological replicates extracted at the weekly time points (0–6 weeks) from wood cultivations, B) for normalized transcript count values of two RNA-sequencing biological replicates from 2 week time point (2 weeks) of malt extract cultivations and 2 and 4 week (4 weeks) time points of wood cultivations, C) hierarchical clustering of the transcriptome samples according to normalized count values. Ellipses in A) represent general trend of the groups with 68 % confidence interval. [file 13068_2016_608_MOESM2_ESM.pdf]
